# Therapeutic non-invasive brain treatments in Alzheimer’s disease: recent advances and challenges

**DOI:** 10.1186/s41232-022-00216-8

**Published:** 2022-10-03

**Authors:** Chongyun Wu, Luoman Yang, Shu Feng, Ling Zhu, Luodan Yang, Timon Cheng-Yi Liu, Rui Duan

**Affiliations:** 1 Laboratory of Regenerative Medicine in Sports Science, School of Physical Education and Sports Science, South China Normal University, Guangzhou, 510006 China; 2 Department of Anesthesiology, Peking University Third Hospital (PUTH), Beijing, 100083 China; 3 Department of Neurology, Louisiana State University Health Sciences Center, 1501 Kings Highway, Shreveport, LA 71103 USA; 4 Department of Neuroscience and Regenerative Medicine, Medical College of Georgia, Augusta University, Augusta, GA 30912 USA

**Keywords:** Alzheimer’s disease, Non-invasive therapy, Photobiomodulation, Transcranial magnetic stimulation, Transcranial direct current stimulation, Exercise

## Abstract

Alzheimer’s disease (AD) is one of the major neurodegenerative diseases and the most common form of dementia. Characterized by the loss of learning, memory, problem-solving, language, and other thinking abilities, AD exerts a detrimental effect on both patients’ and families’ quality of life. Although there have been significant advances in understanding the mechanism underlying the pathogenesis and progression of AD, there is no cure for AD. The failure of numerous molecular targeted pharmacologic clinical trials leads to an emerging research shift toward non-invasive therapies, especially multiple targeted non-invasive treatments. In this paper, we reviewed the advances of the most widely studied non-invasive therapies, including photobiomodulation (PBM), transcranial magnetic stimulation (TMS), transcranial direct current stimulation (tDCS), and exercise therapy. Firstly, we reviewed the pathological changes of AD and the challenges for AD studies. We then introduced these non-invasive therapies and discussed the factors that may affect the effects of these therapies. Additionally, we review the effects of these therapies and the possible mechanisms underlying these effects. Finally, we summarized the challenges of the non-invasive treatments in future AD studies and clinical applications. We concluded that it would be critical to understand the exact underlying mechanisms and find the optimal treatment parameters to improve the translational value of these non-invasive therapies. Moreover, the combined use of non-invasive treatments is also a promising research direction for future studies and sheds light on the future treatment or prevention of AD.

**Supplementary Information:**

The online version contains supplementary material available at 10.1186/s41232-022-00216-8.

## Introduction

First described in 1906 by Dr. Alois Alzheimer, Alzheimer’s disease (AD) has been recognized for more than 100 years [1]. As the most common form of dementia, AD causes progressive memory impairment and is characterized by amyloid plaques and neurofibrillary tangles [2]. There are currently estimated to be over 55 million people living with Alzheimer’s disease or other dementias all over the world, and more than 6.2 million Americans are living with Alzheimer’s disease [3]. Although scientists have made tremendous progress in better understanding the molecular mechanisms underlying amyloid-β and tau pathology in the past decades, the precise mechanisms of AD remain under hot debate, and there are no effective pharmacologic disease-modifying treatments for AD [4].

The failure of molecular targeted pharmacologic therapies has triggered increasing studies to shift toward non-invasive therapies [5]. Non-invasive brain treatment describes treatments for brain diseases that do not require an incision into the brain or tissue removal [6]. In the past decades, non-invasive therapies, including PBM therapy (also known as low-level laser therapy, light therapy), transcranial magnetic stimulation (TMS), transcranial direct current stimulation (tDCS), and exercise therapy, have received increased attention as potential treatments for many brain disorders [7–11]. Although significant advances have been achieved in investigating the effectiveness and underlying mechanisms of these non-invasive therapies, the current understating of the exact underlying mechanism in AD is limited. This review summarizes the pathological changes of Alzheimer’s disease, provides an extensive review of the most widely studied non-invasive approaches, and discusses the primary challenges of these approaches in AD clinical applications.

### Pathological changes in Alzheimer’s disease

#### Amyloid plaques

**Fig. 1:**
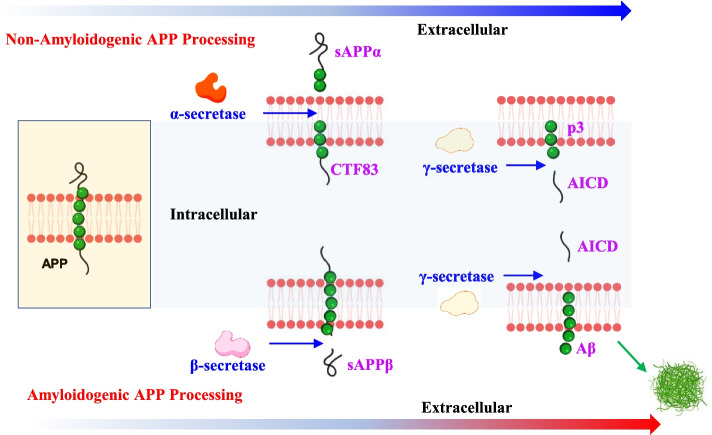
Diagram of amyloid plaque formation. APP, a transmembrane protein, is cleaved by three enzymes, including α, β, and γ-secretase. In the normal physiological state, most of the APP (90% or more) are cleaved by α-secretase and γ-secretase, which generates sAPPα and C terminal fragments (p3, CTF 83, and AICD50). However, under pathological conditions, most of the APP undergoes the amyloidogenic APP processing pathway, wherein Aβ generation is significantly increased and induces the formation of Aβ amyloid fibrils. APP, amyloid precursor protein; sAPPα, soluble amyloid precursor protein α; sAPPβ, soluble amyloid precursor protein β; ACID, APP intracellular domain; CTF, carboxy-terminal fragment

Amyloid plaque is one of the hallmarks of AD [12]. It is formed by the accumulation of extracellular Aβ, a 40-42 amino acid peptide derived from the amyloid precursor protein (APP) [13]. The amyloid hypothesis has been the mainstream explanation for AD’s etiology and pathogenesis since 1991 [12, 14]. As shown in Fig. 1, APP, a transmembrane protein, is cleaved by three enzymes: α, β, and γ-secretase [15]. In the normal physiological state, most of the APP (90% or more) are cleaved by α-secretase and γ-secretase, which generates sAPPα and C terminal fragments (p3, CTF 83, and AICD50). The remaining APP is cleaved by β- and γ-secretase and generates Aβ, which is rapidly removed or degraded [12]. However, under pathological conditions, most of the APP undergoes the amyloidogenic APP processing pathway, wherein Aβ generation is significantly increased and induces the formation of Aβ amyloid fibrils [16]. The accumulation of Aβ induces neurotoxicity, which finally leads to neuronal death and neurodegeneration [16].

#### Neurofibrillary tangles

**Fig. 2:**
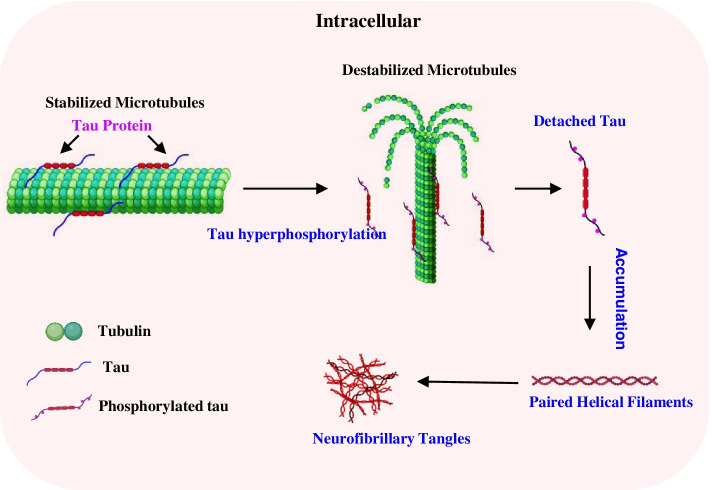
Diagram of the formation of neurofibrillary tangles. Under normal conditions, tau binds to the microtubules facilitating microtubule assembly and promoting microtubule stabilization. In AD, the abnormally phosphorylated tau dissociates from the microtubules, and the phosphorylated tau proteins form neurofibrillary tangles

Neurofibrillary tangles (NFTs) are another hallmark involving the pathogenesis of AD [17]. NFTs are made of abnormally hyperphosphorylated tau, a microtubule-binding protein that maintains the microtubule structure [18]. Microtubules are α and β tubulin subunits-formed hollow cylinder structures [19]. Under normal conditions, tau binds to the microtubules facilitating microtubule assembly and promoting microtubule stabilization [20]. As a significant component of the cytoskeleton, microtubules play a central role in axonal transport, cell motility, and the maintenance of cell shape [19]. In addition, the tau-associated regulation of microtubules is involved in the dynamic control of protein kinases and phosphatases [21]. In AD, the abnormally phosphorylated tau dissociates from the microtubules, and the phosphorylated tau proteins form neurofibrillary tangles (Fig. 2), which results in the interruption of axonal transports and cell signal communication [18].

#### Mitochondrial dysfunction

The mitochondria are essential organelles in eukaryotic cells that carry out multiple essential functions, including the generation of adenosine triphosphate (ATP), intracellular signaling, biosynthesis of neuronal iron-sulfur centers and heme, regulation of cell survival, and apoptosis under various stresses [22–24]. To date, a large body of research has shown that mitochondrial dysfunction and impaired energy metabolism resulting from deleterious fragmentation is an early and causal event in AD [22]. Aberrant mitochondrial fragmentation, resulting from the imbalance of the fusion-fission process, plays a central role in the induction of mitochondrial dysfunction and contributes to the pathogenesis and pathology of AD [22, 23].

**Fig. 3:**
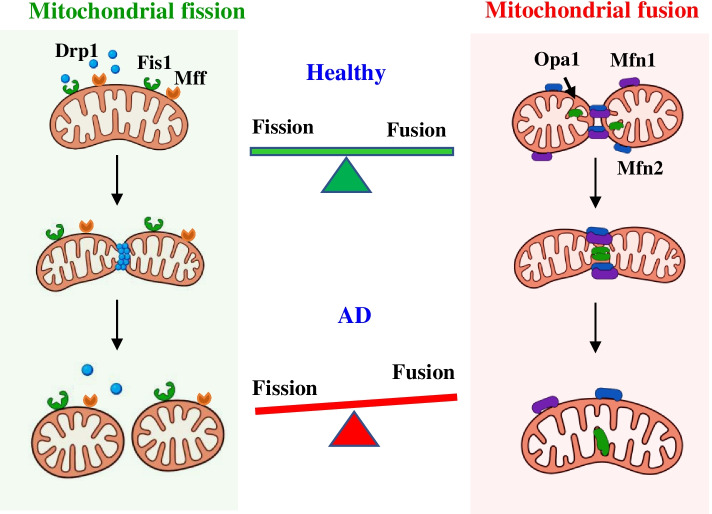
Mitochondrial fission and fusion. Under healthy conditions, the healthy mitochondrial network is maintained by the balance of mitochondria fission (right) and fusion. Drp1 is located in the cytosol, and Fis1 and Mff are located on the outer mitochondrial membrane. Mitochondrial fission occurs when the Fis1 and MFF recruit cytosolic Drp1 to the outer mitochondrial membrane. Mitochondrial fusion is mediated by Mfn1 and Mfn2 from the mitochondrial outer membranes and Opa1 in the mitochondrial inner membranes

In the living cell, mitochondrial morphologies are far from static [25] and undergo specific mitochondrial dynamics involving coordinated cycles of mitochondrial fission and fusion [26]. Mitochondrial fission is essential for generating new mitochondria and mitochondrial quality control (removing damaged mitochondria and mitochondrial apoptosis during high cellular stress levels) [25]. As shown in Fig. 3, mitochondrial fission is mediated by Drp1, Mff, and Fis1 [27]. Drp 1 is located in the cytosol, and Fis1 and Mff are located on the outer mitochondrial membrane [27]. Mitochondrial fission occurs when the Fis1 and MFF recruit cytosolic Drp1 to the outer mitochondrial membrane [25]. In contrast, mitochondrial fusion is a process that joins two mitochondria together, which is vital in protecting mitochondrial integrity [25]. In addition, mitochondria can compensate for other mitochondria’s defects during mitochondrial fusion by sharing components to maintain their integrity [25]. Mitochondrial fusion is mediated by the Mfn1 and Mfn2 from the mitochondrial outer membranes and Opa1 in the mitochondrial inner membranes [22, 25].

Mitochondrial fission and fusion work together to preserve mitochondrial health and function [25, 28]. Mitochondrial fission segregates the most severely damaged mitochondria and removes them through quality control, while mitochondrial fusion corrects low levels of damage by sharing components [25, 29]. In the AD brain, the balance between mitochondrial fission and fusion is disrupted by significantly increased fission protein expression and decreased fusion protein expression, which induces excessive mitochondrial fragmentation in vulnerable neurons (Fig. 3) [7, 30, 31].

**Fig. 4:**
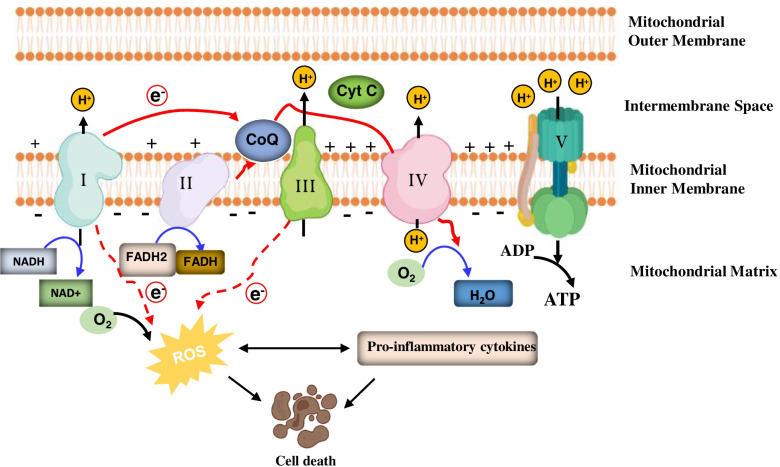
Generation of reactive oxygen species (ROS) by the mitochondria. Under physiological conditions, a small number of electrons (dotted red line) leak out of the ETC and are transferred to oxygen to produce reactive oxygen species (ROS). However, in pathological situations, mitochondrial dysfunction and the impaired mitochondrial complex activity induce significantly increased ROS production. This leads to deleterious effects and a vicious cycle causing neuroinflammation, mitochondrial damage, energy depletion, neuronal damage, and cell death

In addition to abnormal mitochondrial dynamics, the role of mitochondrial bioenergetic deficits in AD has been well established [32, 33]. The high energy demand of neuronal cells suggests that mitochondrial bioenergetic deficits contribute to neuronal death in AD [34]. There are five multiprotein enzyme complexes in the mitochondrial inner membrane involved in ATP production: complex I (NADH-ubiquinone oxidoreductase), complex II (succinate-ubiquinone oxidoreductase), complex III (cytochrome bc1 complex), complex IV (cytochrome c oxidase), and complex V (ATP synthase) (Fig. 4) [22]. These five enzyme complexes’ reduced expression and activity have been found in the brain of AD patients and animals, suggesting mitochondrial bioenergetic deficit precedes Alzheimer’s pathology [32, 35, 36].

#### Oxidative stress

Compelling evidence has demonstrated the significantly increased oxidative stress in AD brains [37]. The imbalance between free radical production and antioxidant defenses in AD induces excessive oxidative stress in the AD brains [37]. As the “powerhouses of the cell,” mitochondria are also the primary intracellular source of oxygen radicals under both physiological and pathological conditions [22, 23]. As shown in Fig. 4, under physiological conditions, a small number of electrons leak out of the electron transport chain (ETC) and are transferred to oxygen to produce reactive oxygen species (ROS) [38]. The ROS generated serves as second messengers in mediating several critical intracellular pathways [39, 40]. In pathological situations, mitochondrial dysfunction and the impaired mitochondrial complex activity induce significantly increased ROS production. This leads to deleterious effects and a vicious cycle causing mitochondrial damage, energy depletion, neuronal damage, and cell death (Fig. 4) [41, 42]. Several studies demonstrate the increase in oxidative damage to DNA, proteins (protein carbonyl), and lipids (lipid peroxidation), which contribute to the initiation and progression of AD [43–45].

#### Neuroinflammation, polarization of glial cells, and microglial phagocytosis

**Fig. 5:**
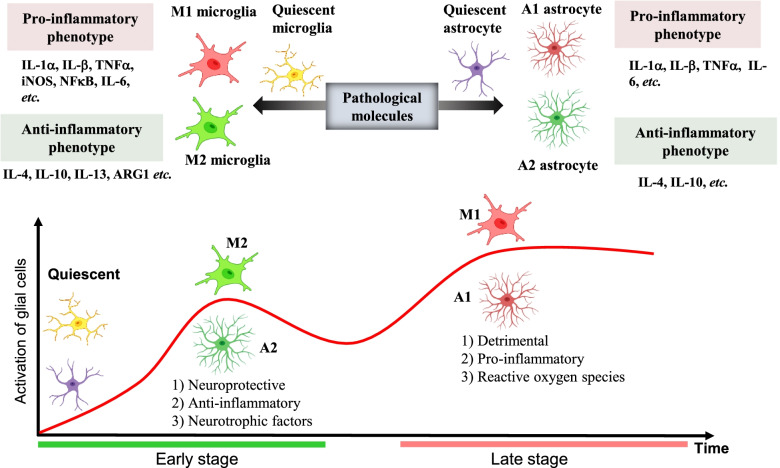
Activation and polarization of microglia in AD. The M1 phenotype produces pro-inflammatory cytokines (i.e., IL-1β, TNF-α, NFκB, and IL-6), exacerbating AD progression. However, the M2 phenotype release anti-inflammatory cytokines (i.e., IL-4, IL-10, and IL-13), providing neuroprotective effects in AD. Similar to microglia, astrocytes are classified into A1 neurotoxic and A2 neurotrophic/neuroprotective phenotypes. In the early stage of AD, quiescent glial cells are activated to the A2 and M2 phenotypes and transformed into A1 and M1 phenotypes at the late stage of AD transformation

Neuroinflammation triggered by pathological molecules including Aβ, tau, and damage-associated molecular patterns (DAMPs) in AD has been well demonstrated [44, 46]. In the early stages of AD, the deposition of Aβ acutely initiates the activation of microglia to remove Aβ through phagocytosis [47]. During this stage, the activation of microglia works as a protective response against AD [48]. However, microglial phagocytosis fails to remove amyloid plaques, and the increasing microglial activation releases an array of pro-inflammatory mediators that contribute to AD’s progression [49]. As shown in Fig. 5, the activated microglia can be divided into pro-inflammatory subtype M1 and M2 anti-inflammatory phenotype [50]. The M1 phenotype produces pro-inflammatory cytokines (i.e., IL-1β, TNF-α, IL-6, and IL-12), exacerbating AD progression. However, the M2 phenotype release anti-inflammatory cytokines (i.e., IL-4, IL-10, IL-13, and TGF-β), providing neuroprotective effects in AD [51, 52]. Furthermore, the M1 phenotype display relatively poor phagocytosis of Aβ, and the M2 phenotype shows elevated phagocytosis [53, 54]. Taken together, in the early stage of AD, the quiescent microglia are polarized into the M2 phenotype to confer a neuroprotective effect by releasing anti-inflammatory and neurotrophic factors. At the late stage, the microglia are polarized into the M1 phenotype, inducing neuronal loss and exacerbating AD progression by releasing pro-inflammatory cytokines and ROS (Fig. 5) [51]. Recently, a study using single-cell RNA-seq in the AD animal models identified a novel microglia type called disease-associated microglia (DAM), which represent a distinctive microglia states detected in AD but not in the wild type brains. The DAM coexists with the homeostatic microglia and infiltrating monocytes. Similarly, the transition between the homeostatic microglia to the DAM subtype is consistent with the direction of AD progression [55, 56].

Similar to microglia, the activation of astrocyte, another sub-type of glial cells in the central nervous system, is also found near the sites of amyloid plaques [57]. In postmortem tissues from both AD patients and animal models, astrogliosis is observed and correlated with cognitive decline in AD [58–60]. According to recent studies, astrocytes are classified into A1 neurotoxic phenotype and A2 neurotrophic/neuroprotective phenotype [61, 62]. A1 phenotype can be activated by factors and fragmented mitochondria released from microglia to trigger and exacerbate neuroinflammation in AD [63, 64]. In contrast, the A2 phenotype is proposed to be a reparative and neuroprotective astrocyte phenotype in the brain [65]. According to previous studies, the phenotypical switch from the A1 phenotype to the A2 phenotype ameliorates AD pathology [66, 67].

A previous study showed that young microglia could restore the capacity of amyloid plaque clearance of aged microglia [68], and approaches that trigger the recruitment of microglia around amyloid plaques display a potential effect on attenuating AD pathology [69, 70]. Recently, the crosstalk between astrocytes and microglia provides a novel mechanism for the microglial recruitment around amyloid plaques [71]. In both the humans and mice, the astrocytic interleukin-3 (IL-3) can target the microglial IL-3 receptor (IL-3R) to induce the recruitment of microglia and enhance the ability to clear Aβ and tau, suggesting the astrocytic IL-3 is a crucial mediator of microglial recruitment and a potential target for the regulation of microglial phagocytosis [71].

#### Challenges for AD studies

Currently, no preventive or curative treatment is established to be safe and efficacious for AD [72]. Almost all molecular targeted pharmacologic therapies developed to treat or slow down AD have failed in clinical trials [73]. Although scientific advances in the past few decades have expanded our understanding of AD’s cellular and molecular basis, the exact mechanisms of AD generation remain to be fully unveiled [74]. Additionally, for AD patients, the pathophysiological changes of AD precede the clinical symptoms for many years [74]. For example, prominent deposition of amyloid plaques has already been displayed in the brain tissue before memory loss, which is difficult to be reversed [75]. Therefore, finding early detection biomarkers will be one of the primary research focuses of AD in future studies [74]. In addition, currently, nearly all the pharmacologic therapies in AD are based on the “lock-and-key” model, and the discovery of drugs focuses only on a single target (e.g., Aβ or tau) [76]. However, AD develops from various factors [77]. Therefore, further studies focusing on multiple targets may help facilitate AD treatment and prevention [76]. Moreover, experimental animal models are essential for better understanding of AD pathogenesis and progression and performing a preclinical assessment of the potential novel therapeutics [78]. To date, transgenic mice that express mutant forms of human APP, tau, and/or presenilin-1 (PS1) are considered as the “gold standard” animal models of AD [79]. However, these transgenic mice only have limited aspects of AD pathologies of humans, which hampers the successful transmission of animal experiments to clinical trials [78]. Therefore, choosing an appropriate animal model is essential for future AD studies.

### PBM therapy and parameters of intervention

PBM therapy, also known as low-level laser (light) therapy, is a non-invasive photoceutical approach involving the application of relatively low levels of visible (wavelength between 400 and 720) or near-infrared light (wavelength between ~700 and 100 nm) on biological tissues to improve healing, relieve inflammation and pain, and preserve tissue function [22, 80]. As a non-invasive treatment method, PBM therapy describes the application of the low-level laser (light) directly to a specific region of interest on the body to modulate various biological processes [6]. It was first discovered by Endre Mester, a Hungarian physician working with wound healing and hair regrowth in 1967 [81, 82]. He found quicker hair growth and wound healing in the rats with low-level laser treatment [81, 82], which was the first study that found the beneficial application of low-level laser light on biological processes [83]. Since then, an increasing paper has described this treatment as “laser biostimulation,” “low-level laser (light) therapy (LLLT),” and now “PBM therapy” [84–86]. Although PBM therapy was initially applied to promote hair regrowth and wound healing [81, 82], the beneficial effects of PBM on relieving pain and inflammation and promoting muscle recovery have been widely studied [87–90]. In the past decades, the potential role of PBM therapy in the treatment of brain disorders has garnered increasing attention [7, 8, 91, 92]. Recent studies provide considerable evidence of PBM’s promising therapeutic potential in AD treatment [7, 69, 70].

Multiple parameters simultaneously affect the efficiency of PBM therapy, including wavelengths, intensities, durations, target area, and operation mode. Currently, the most widely used wavelength is red (~600–700 nm) and near-infrared wavelengths (~780–1100 nm) [93]. For the PBM therapy with wavelength within 600–1100, the primary target is the cytochrome c oxidase (CCO), the unite IV of the mitochondrial respiratory chain [22]. Additionally, evidence supports that blue and green light with shorter wavelengths within 450–570 nm can also confer PBM effects [94]. The effects may rely on intracellular calcium and light-gated ion channels [94]. However, the low transmission tissue limits the application of blue or green light within 450–570 nm [94].

Another critical parameter of PBM is the dose of the light source. The treatment doses of PBM depend on the intensities, duration, and PBM targeted area [93, 95, 96]. Currently, there is no consensus about treatment doses of PBM therapy. Except for the efficiency of the PBM therapy, the primary concern for the treatment dose of PBM is the thermal effects [97]. The intensities that produce the unacceptable thermal impact of the tissue are ~300 mW/cm^2^ at 600 to 700 nm, about 750 mW/cm^2^ at 800 to 900 nm, and 100 mW/cm^2^ at 400–500 nm [96]. Furthermore, the exposure duration is also critical for the efficiency of PBM therapy [96, 98]. Studies showed that even a few minutes of PBM therapy could cause biological changes [99, 100]. However, the best exposure duration also relies on other parameters used in the PBM application [96].

Aside from these parameters, the mode of operation of PBM can either be a pulsed wave or a continuous wave [101]. Both pulsed and continuous wave PBM display beneficial effects in AD [7, 69, 70, 102]. The pulsed wave PBM refers to the PBM effect induced by the light source in pulses of some duration at some repetition rate [103]. The most widely studied frequency is 10 Hz or 40 Hz in AD [69, 70]. Although different operation mode of PBM has similar effects, the underlying mechanism differs, which will be discussed in the following section.

### PBM therapy for AD

#### Improves behavioral results and reduces amyloid plaques and neurofibrillary tangles

Intriguingly, increasing human and animal studies suggest that PBM treatment is a promising potential therapy in AD [69, 70, 104–107]. Progressive memory impairment is one of the primary signs of AD and is usually the first and common complaint for the patient to seek diagnosis [7, 108, 109]. As a hallmark of AD, memory impairment/loss is one of the most common clinically relevant markers to assess the effect of a potential treatment [110]. In a previous study, the learning and memory deficits were significantly improved by PBM at 40 Hz (light-emitting diode) in the AD mouse model [70]. The non-invasive 40-Hz light also reduced the levels of Aβ1-40 and Aβ1-42, regulated microglia’s morphological transformation, and improved both the phagocytosis and migration/cell adhesion-related genes in the cortex and hippocampus, which helped the improvement of cognitive function [70]. In a recent study, PBM with 1070-nm pulsed-wave light at 10 Hz could also improve learning and memory impairments after PBM treatment in AD mice [69]. Moreover, the AD animals with PBM therapy displayed reduced Aβ burden and improved Aβ clearance by regulating microglia and angiogenesis [69]. Another study found that PBM could promote the permeability of BBB, which results in increased Aβ leakage followed by further activation of the lymphatic clearance of Aβ [111]. As shown in Supplementary Table 1, the PBM has been applied to the frontal cortex, temporal regions, base of the skull, wrist, nose cavity, abdomen, and forehead bilaterally or generated gamma entrainment using sensory stimuli through the eyes in human clinical trials. Currently, most PBM-associated clinical trials are ongoing or terminated due to the coronavirus. Therefore, whether PBM can reduce the amyloid plaques in the whole brain is unclear. However, according to previous animal studies, the PBM therapy can alleviate at least amyloid plaques in the cortex and hippocampus [70, 112]. These findings added more evidence to the therapeutic effect of pulsed-mode PBM.

In addition to PBM with pulsed wave light, several studies identified the beneficial role of PBM with continuous wave light [7, 102]. In an Aβ-induced AD rat model, PBM treatment with the continuous-wave laser diode at 808 nm for 5 days protected against Aβ-induced cell toxicity and long-term spatial and object recognition memory [102]. In addition, the 5-day PBM treatment alleviated the hyperphosphorylated tau (PHF1) protein expression and neuronal apoptosis [102]. These findings were consistent with another research that found that PBM treatment with the continuous-wave laser at 632.8 nm suppressed neuronal loss and dendritic atrophy in the APP/PS1 double-transgenic AD mouse model [113]. Furthermore, anxious-depressive-like behavior has been detected and recognized as an early sign of AD pathogenesis [7]. Growing evidence implied that early treatment of anxious-depressive-like behavior could lower the risk of developing AD [7, 114]. The beneficial effect of PBM treatment in alleviating depression and anxiety-like behaviors has been widely reported [92, 115–117]. Intriguingly, PBM treatment with a continuous-wave laser could attenuate anxious-depressive-like behavior and protect against neuronal damage and apoptosis in the AD rat animal model, supporting the potential role of PBM therapy in preventing or slowing down the progression of AD [7].

Moreover, the beneficial effect of PBM on AD has also been identified by numerous clinical trials [104–107]. Using an 810-nm, 10-Hz pulsed LED light source PBM therapy, AD patients with moderate-to-severe cognitive impairment were assigned to receive 12-week PBM treatment. Results showed that 12 weeks of active PBM treatment significantly improved AD patients’ cognitive function and reduced anxiety [107]. Another clinical trial with continuous-wave near-infrared PBM therapy concluded that in PBM treatment patients displayed better cerebral perfusion and resting-state functional connectivity and a significantly improved cognitive and behavioral function [105]. Moreover, a case report further supported these previous observations [118]. In this case report, patients diagnosed with both cognitive decline and olfactory dysfunction received a mixture of continuous-wave mode red (635 nm), near-infrared light (NIR) LEDs (810 nm), and 10-Hz pulsed wave mode NIR (810 nm) PBM therapy. After PBM therapy, significant improvements were detected in the Montreal Cognitive Assessment and Working Memory Questionnaire [118]. As shown in Supplementary Table 1, an increasing number of clinical trials are testing non-invasive therapy in AD. Among these clinical trials, a clinical trial involving gamma entrainment therapies (also known as gamma entrainment using sensory stimuli, or GENUS) based on previous studies has entered phase II clinical trials in AD patients. Previous studies found that gamma entrainment therapies alleviate cognitive deficits and improve the clearance of amyloid plaques by recruiting neuronal and glial responses, shifting neurons to a less degenerative state, releasing neuroprotective factors, enhancing synaptic function, alleviating neuroinflammation, and reducing DNA damage-associated cytotoxicity in neurons [70, 112]. In these studies, 40-Hz pulsed light or 40-Hz blue light with 40-Hz auditory stimulation was employed to produce GENUS and exhibited neuroprotective effects in the AD mouse model, suggesting an alternate form of PBM in treating neurodegenerative diseases. Overall, these findings support the potential therapeutic application of PBM therapy in improving cognitive impairment and reducing anxious-depressive-like behaviors of AD.

#### Preserves mitochondrial function and structure

**Fig. 6:**
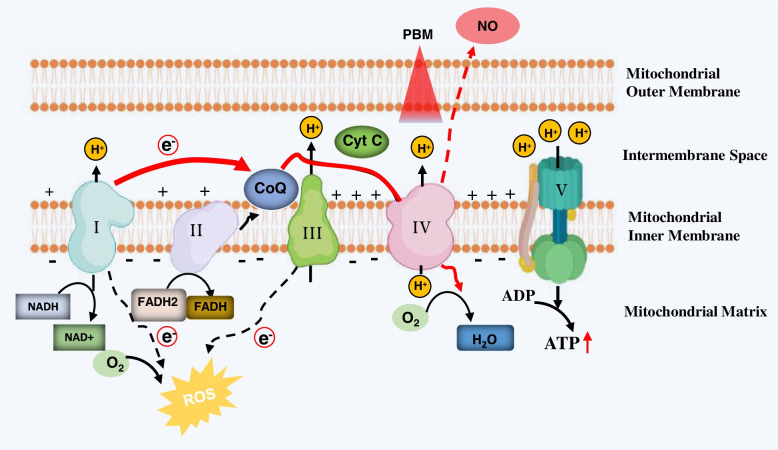
PBM promotes CCO activity and increases ATP production. Nitric oxide (NO) inhibits CCO activity by non-covalently binding with CCO. PBM treatment leads to the dissociation of NO from CCO, causing the increased activity of this complex and ATP production

As mentioned previously, mitochondrial dysfunction and aberrant mitochondrial fragmentation are involved in the occurrence and development of AD and are recognized as common features of neurodegenerative disease [22]. It is widely accepted that mitochondrial cytochrome c oxidase (CCO, complex IV of the respiratory chain) is the primary action site of PBM therapy [8, 22]. Therefore, a large body of evidence suggests that the beneficial effect of PBM therapy on AD is mainly due to the regulation of mitochondrial function and mitochondria-related processes [8, 22]. The primary mechanism underlying PBM’s regulation of mitochondria is involved in the modulation of CCO activity [119–122]. Nitric oxide (NO), a molecule that binds non-covalently to the heme iron and copper centers of CCO to inhibit the activity, is the most important medium in this process [119–122]. PBM therapy enhances CCO activity by photodissociating NO from CCO, thereby reversing the inhibition of the electron transport chain due to excessive NO binding (Fig. 6) [123, 124]. The effect of PBM on CCO indicates that PBM treatment is a potential intervention for promoting mitochondrial function in AD [123].

Besides the direct effect on mitochondrial CCO, PBM therapy regulates mitochondrial dynamic and fragmentation [125, 126]. The balance of mitochondria fission and fusion is crucial for the normal function of mitochondria and the maintenance of mitochondrial morphology [22, 127]. Aberrant or increased mitochondrial fission in AD leads to increased mitochondrial fragmentation and neuronal death [128]. Intriguingly, several previous studies have demonstrated that PBM treatment preserved the dynamic equilibrium between mitochondrial fusion and fission in various brain diseases such as global cerebral ischemia, neonatal hypoxic ischemia, AD, and Parkinson’s disease [8, 125, 129, 130]. In an Aβ1-42-induced AD rat model, PBM treatment with continuous-wave low-level diode laser significantly alleviated excessive mitochondrial fission-induced mitochondrial fragmentation by promoting mitochondrial fusion-related proteins (OPA1 and MFN1) and inhibiting mitochondrial fission-related protein (Drp1, Fis1, Mff, and Mief) [102]. Similar regulation of these proteins was also observed in other brain disease models [8, 22, 119]. Taken together, PBM, a mitochondria-targeted therapy, demonstrated its therapeutic potential in AD treatment by regulating the mitochondrial structure and mitochondrial function.

#### Regulates glial cells and exerts anti-inflammatory effects

Emerging evidence from previous studies demonstrated the beneficial role of PBM therapy in regulating glial cells and neuroinflammation [8, 69, 70, 121, 131]. In a previous study, 40-Hz light flicker PBM therapy recruited microglia around amyloid plaques and improved microglial phagocytosis and migration/cell adhesion-related genes in multiple mouse models [70]. The modification of microglia after PBM treatment triggers microglia to increase Aβ uptake [70], indicating the potential role of PBM treatment in improving microglial phagocytosis. Recently, results from another study further our understanding of PBM treatment on the regulation of glial cells [69]. As reported in their work, PBM treatment with 1070-nm pulsed-wave light at 10 Hz can also reduce cerebral Aβ burden by promoting the activation of microglia (e.g., increased cell body, reduced number and length of branches) and microglial phagocytosis [69]. Notably, they found a decreased pro-inflammatory M1 phenotype and the increased M2 anti-inflammatory phenotype after PBM treatment [69]. These findings demonstrate that PBM can inhibit neuroinflammation by promoting the transformation of microglia from a neurotoxic to a neuroprotective phenotype in AD [69]. Anti-inflammatory effects were also found in PBM therapy using continuous-wave lasers [102]. In the Aβ1-42-induced AD rat model, Aβ injection into the hippocampus of rats led to the increased release of pro-inflammatory cytokines (i.e., IL-1β, IL-5, and TNF-α), mitochondrial dysfunction, demyelination, and axonal damage of neurons [102, 132, 133]. Interestingly, PBM treatment with continuous-wave low-level diode laser significantly suppressed Aβ-induced neuroinflammation and protected against Aβ-induced neuronal injury and neurodegeneration [102]. However, in this AD rat model, PBM treatment with continuous-wave lasers significantly suppressed Aβ-induced reactive gliosis [102], which is different from the pulsed-wave PBM’s promoting the activation of glial cells [102]. One of the explanations for the difference may be due to the animal model. Aβ-induced AD-like rat model is an acute AD rat model, wherein Aβ induces reactive gliosis rapidly. However, in the transgenic mouse model, glial cells are activated progressively. PBM inhibits glial cells’ acute activation to protect against inflammatory response-induced neuronal damage in the acute AD animal models but promotes the activation and polarization of glial cells to a neuroprotective phenotype in the progressively developed AD models [51, 102].

#### Inhibits oxidative stress and oxidative damage

The anti-oxidative effect of PBM has been widely studied in the skeletal muscle after physical exercise [134–136]. Oxidative stress is also implicated in AD’s pathogenesis and progression [37, 137]. The significantly elevated levels of 4-hydroxyhexenal (4HHE), a lipid peroxidation marker, have been found in animal models and in vitro cell cultures [44, 138]. Moreover, the level of 8-hydroxydeoxyguanosine (8-OHdG), a widely used DNA oxidative marker, was a threefold increase in the postmortem brain tissue of AD patients compared with age-matched controls [139]. Similar results were found in AD-like animal models [44, 140]. Furthermore, the oxidative damage of protein induced by excessive free radicals was detected in sporadic AD rat and transgenic AD models [7, 44, 141]. Additionally, several enzymes critical to neuron and glial functions are prone to oxidative damage and decrease in AD [37]. For example, the enzymes susceptible to oxidative stress, the glutamine synthetase and creatine kinase, are significantly reduced in AD brains, inducing decreased glutamate concentrations and excitotoxicity enhancement [37, 141]. Moreover, oxidative stress impaired creatine kinase activity and caused reduced energy metabolism in AD [37, 141]. Interestingly, PBM with different light sources and parameters shows its significant anti-oxidative effect in neuronal cell culture and AD brains. PBM treatment with 660-nm continuous-wave LED at 20 mW/cm^2^ protects against neuronal cell death by reducing H_2_O_2_-induced oxidative stress in vitro study [142], wherein the improved antioxidant enzyme and redox homeostasis are considered as the key mechanism underlying this protection [142]. Further evidence from two transgenic mouse models (APP/PS1 and K369I tau transgenic model) supports the anti-oxidative effect of 670-nm near-infrared light [143]. After receiving 90-s PBM treatment for 5 days per week, the AD animal displayed a significant reduction in neurofibrillary tangles, hyperphosphorylated tau, and oxidative stress markers (4-HNE and 8-OHdG) in the cortex and hippocampus [143].

The anti-oxidative effect of PBM is closely related to the preservation of mitochondrial function [22]. As mentioned above, the primary action sit of PBM therapy is CCO [8, 22]. A short burst of ROS is generated when NO is photodissociated from CCO after PBM treatment [8, 22]. The temporary and relatively modest increase of ROS production leads to the activation of the NF-κB and PI3K/Akt pathway [125, 144, 145]. The activation of NF-κB will lead to the translocation from the cytoplasm to the nucleus. It induces more than 150 gene expressions, including genes associated with antioxidant activity and mitochondrial dynamics [22, 146]. Therefore, oxidative stress is significantly alleviated in the cells subjected to stress with PBM treatment [147]. This evidence explains how PBM therapy reduces clinical oxidative stress and protected neurons from death in various lesions and AD [147].

### Challenges of PBM therapy in AD

Although the beneficial effects of PBM therapy have been found in different tissues and widely studied in brain disease, challenges remain in the clinical application of PBM on AD patients [83]. First, there is no agreement on the parameters and protocols of PBM therapy in the clinical application of AD [96]. As mentioned previously, several light source parameters are involved in the effects of PBM therapy. The prevailing use of a wide variety of light sources, the illumination parameters (e.g., wavelength, power density, pulse structure, fluence), and treatment protocols (a single application of light or multiple doses) induce significant variations in the study design [83, 98]. The disagreement on light/laser parameters and the variations in study designs led to many negative results in clinical trials and posed some controversy in PBM’s study and application [83]. Second, although CCO has been widely accepted as the primary target of PBM, there is still conflicting data doubting whether it is the primary acceptor [148, 149]. For example, a study found that PBM could promote ATP production and increase cell proliferation in CCO knockout cells, suggesting that CCO may not be the primary or only target of PBM therapy [149]. Therefore, more studies are still needed for the precise mechanisms of PBM treatment in AD. Although increasing evidence supported the beneficial effect of pulsed-wave PBM therapy, the exact mechanisms of pulsed-wave PBM therapy in AD remain elusive [69, 70]. Finally, in animal studies and clinical trials, almost all studies performed PBM directly at the target tissue. However, increasing studies over the past several years demonstrated that the beneficial effects of PBM therapy are not limited to irradiated tissue [150, 151]. However, the indirect or remote PBM therapy mechanisms remain to be understood. More studies on the remote PBM therapy have far-reaching implications on the therapeutic application of PBM in AD as the brain is a difficult-to-irradiate organ [150, 151].

### Transcranial magnetic stimulation and parameters of intervention

Transcranial magnetic stimulation (TMS) is another non-invasive approach developed to treat multiple neurodegenerative diseases such as AD [152, 153]. TMS relies on electromagnetic pulses to stimulate brain cells wherein the changing electric-induced magnetic field is applied to a target region of the brain [10, 154]. Generally, the apparatus used for TMS is composed of an electric pulse generator (stimulator) and a magnetic coil [155]. The electric pulse generator is connected to the magnetic coil, which produces a magnetic field delivering non-invasive magnetic pulses to a specific brain area at different intervals and frequencies [155]. The physical principles of TMS were first described by Michael Faraday, an English physicist, in 1881 [156]. Then the magnetic field targeting a specific brain was found to induce physiological and behavioral changes [156, 157]. After that, Anthony Barker and his team first introduced the TMS device and stimulated the brain’s neurons in the left cerebral motor strip to move the right hand, which was the first time the TMS was used to direct physical response and also a demonstration of TMS being capable of non-invasively stimulating a specific area of the brain without causing pain which is a case for electrical stimulation [158]. Currently, the repetitive transcranial magnetic stimulation (rTMS) delivering repetitive magnetic pulses has been adopted into clinical practice for the treatment of major depressive disorder (MDD) [159], and now when people mention the TMS therapy, it usually refers to rTMS [159]. The development of rTMS as an antidepressant therapy has been supported by multiple randomized controlled trials and published literature [160–162]. In the clinical trials, patients with rTMS treatment showed significant improvement in the depressive symptoms and higher rates of remission [160–164]. Therefore, since the US Food and Drug Administration (FDA) approved the first device for rTMS treatment of MDD in 2008, FDA has cleared 5 TMS devices for MDD treatment in the USA [160]. The safety and efficacy of rTMS in anti-depression drives people’s interest in other neurological diseases.

Similar to PBM, the effect of rTMS relies on several parameters, including interstimulus interval, stimulus intensity, number of stimuli, the interval between successive trains, stimulus duration, and delivery mode [165]. The long-lasting after-effect triggered by rTMS treatment depends on the combinations of different parameters [166]. In general, the operation of rTMS can be divided into “low or high frequency” and “conventional or patterned TMS” [165, 166]. Low-frequency stimulation and high-frequency refer to stimulation rates lower than 1 Hz and higher than 5 Hz, respectively [165, 166]. In most low-frequency rTMS studies, the low-frequency therapy induced an inhibitory effect on cortical excitability, and high-frequency rTMS led to an excitatory effect [166]. rTMS with 10 Hz and 20 Hz is the most commonly used frequency in AD clinical trials [166–168]. The conventional TMS refers to a single TMS pulse application in a regular rhythm, and the patterned rTMS refers to the application of TMS with short, high-frequency bursts with brief periods of no stimulation [166]. Increasing evidence supports the beneficial effects of theta-burst stimulation (TBS) on AD recently [152, 169]. TBS is a typically patterned rTMS, wherein short bursts of high-frequency pulses repeated at 5 Hz are delivered based on the natural theta rhythm in the hippocampus [152, 166]. In terms of delivery modes, the TBS can be further divided into continuous TBS (uninterrupted sequence) and intermittent TBS [166, 170]. As mentioned above, beyond stimulation frequency and delivery mode, stimulus intensity and duration are important factors influencing the outcome of rTMS. However, unfortunately, there is no consensus on the optimal stimulation protocols [165].

### rTMS therapy for AD

#### Improves cognitive function of AD and decreases Aβ accumulation and tauopathy

Increasing evidence from clinical and animal studies supported rTMS as a promising treatment for mild and moderate AD [171–173]. At the early stages of AD, high-frequency rTMS treatment with 40 burst trains at 20 Hz was delivered to the head of a transgenic familial AD mouse model (5xFAD mice). After 14 consecutive days of rTMS treatment, AD animals with rTMS displayed significant improvement in long-term memory performance, suggesting the beneficial effects of rTMS with 20 Hz for memory impairment [171]. Similar to high-frequency rTMS treatment, relatively low-frequency rTMS treatment also plays a beneficial role in improving cognitive impairment [174]. Furthermore, rTMS at 5 Hz delivered to the head for 14 consecutive days significantly also enhanced the learning and memory of APP/PS1 mice, as evidenced by shortened escape latency and increased time in the target quadrant in the AD-like mouse model rTMS treatment [174]. Consistent with the behavioral results, rTMS at 5 Hz reduced neuronal Aβ accumulation and tau hyperphosphorylation, the hallmark pathologies in AD progress [174, 175]. The underlying mechanisms include reducing Apolipoprotein E (ApoE) expression and promoting autophagic flux following rTMS treatment, which is in line with a previous finding that rTMS promotes the BBB-mediated drainage efficiency of the brain clearance pathways in an AD animal model [171, 174].

Mild cognitive impairment (MCI) is one of the typical symptoms of AD [176]. In a scopolamine-induced AD-related MCI mouse model, both the low- and high-frequency rTMS (1 Hz and 10 Hz) were applied to the head of the rats for two sessions. AD mouse with rTMS treatment showed significantly improved cognitive function in a frequency-dependent manner, with the high-frequency rTMS exhibiting better improvement effects on cognitive function [176]. Similar results were found in the Aβ42 administration-induced AD mouse model [177]. Both the low-frequency (1 Hz) and high-frequency (20 Hz) rTMS were able to enhance spatial working memory in a frequency-dependent manner [177], suggesting rTMS therapy exists the optimal dosage, which needs more study. Notably, the effects of rTMS do not only rely on the frequency [178]. It also depends on the intensity of rTMS [178]. The low frequency of rTMS at high intensity appeared to be detrimental to cognitive function [178].

Clinical trials confirmed the effects of rTMS on the cognitive function of AD [175, 179]. For example, AD patients who received high-frequency rTMS at 20 Hz for 2 weeks showed improved auditory sentence comprehension, suggesting that rTMS could modulate short-and/or long-range cortical synaptic efficacy and connectivity, leading to more effective processing [175]. This effect is also found in another rTMS clinical trial with 20 Hz for 6 weeks, in which more measurements were performed to measure cognitive function, including Assessment Scale-cognitive subscale (ADAS-cog), Montreal Cognitive Assessment (MoCA), Mini-Mental State Examination (MMSE), and the World Health Organization-University of California-Los Angeles Auditory Verbal Learning Test (WHO-UCLA AVLT) [179]. In these tests, the MMSE, ADAS-cog, and WHO-UCLA AVLT scores in the rTMS group were significantly improved, suggesting that rTMS is also a promising therapy for enhancing cognitive function in AD patients [179].

#### Promotes synaptic plasticity and hippocampal neurogenesis

rTMS modulates neuronal processing by inducing the depolarization of neural cell membrane potentials under the magnetic field and affecting the related nerve loop activity with prolonged effects on neural activity [154, 180]. The regulation of synaptic plasticity is the most widely accepted mechanism of rTMS [178]. It modulates long-term potentiation/depression (LTP/LTD) of excitatory synaptic transmission and influences spatial cognition [178]. rTMS-induced LTP or LTD depends on the strength of Ca^2+^ internal flow and the intracellular Ca^2+^ level in the postsynaptic membrane, which is determined by the combinations of the rTMS’s parameters [181]. In both the vascular dementia animal model and normal aging model, low-frequency rTMS (1 Hz) at low intensity alleviates cognitive impairment by increasing the expressions of synaptic protein markers and activating brain-derived neurotrophic factor (BDNF)/tropomyosin-related kinase B (TrkB), the key mediators of neuronal and synaptic maturation [178, 182].

Besides the changes in synapses, neuronal loss is another of the hallmarks of AD [183]. Neurogenesis is abundant in healthy individuals [184]. However, the impaired adult hippocampal neurogenesis in AD patients exacerbates neuronal loss and contributes to AD progression [184]. Intriguingly, studies detected neurogenesis after rTMS treatment [178, 185–187]. In a focal cerebral ischemia animal model, rTMS treatment at both low frequency (1 Hz) and high frequency (20 Hz) was analyzed [185]. Notably, high-frequency rTMS at 20 Hz significantly improved neurogenesis in the ischemic striatum [185]. However, neurogenesis was not markedly elevated by rTMS at 1 Hz [185]. Consistent with these findings, the infarct volume was decreased, and functional recovery was enhanced after high-frequency rTMS treatment [185]. In contrast, the neurogenesis was suppressed in the chronic psychosocial stress animal model after 18 days of rTMS treatment at the same frequency [185]. The difference in the other parameters of rTMS, including the intensity and treatment duration, may explain the discrepancies between these two studies. Furthermore, the different animal models may be another possible reason for the different neurobiological effects [187]. A previous study confirmed one of the explanations that intensity may affect the neurobiological effects of rTMS [188]. In their study, rTMS with the same frequency but different intensities was applied on a depressive-like model [188], wherein only medium-intensity rTMS at 50 mT increased BDNF and neurogenesis [188]. Consistent with the findings in the brain injury model, rTMS with intermittent gamma burst stimulation (30–40 Hz) can promote neurogenesis and differentiation of newborn cells into mature neurons in the hippocampus [186]. Although discrepancies exist in the neurogenesis after rTMS treatment in different animal models, increasing evidence suggests rTMS with appropriate parameters can promote neurogenesis [189–191]. More studies on neurogenesis and synaptic plasticity after rTMS are still needed in AD.

#### Neurotransmitters contribute to the beneficial effects of rTMS

The regulation of neurotransmitters is another possible mechanism contributing to the beneficial effects of rTMS [154]. Anxious-depressive-like behavior has been recognized as an early sign of AD [7, 9, 192, 193]. Furthermore, increasing evidence indicated that treatment of depressive-like behavior could attenuate later cognitive deficits, thereby presenting a target to slow AD development [114]. Therefore, the regulation of neurotransmitters associated with anxious-depressive-like behavior and cognitive function contributes to the beneficial effects of rTMS stimulation [154].

Serotonin (5-HT) is an essential excitatory transmitter playing a critical role in neuropsychiatric disorders and memory loss [194, 195]. However, the cortical 5-HT and 5-HT receptor levels were reduced in the postmortem brain tissue of AD patients and patients with major depressive disorder [196–198]. Interestingly, high-frequency rTMS at 10 Hz applied to the left dorsolateral prefrontal cortex (DLPFC) significantly upregulates 5-HT content and 5-HT receptors [199–202]. Similar results are also found after treatment with low-frequency rTMS at 1 Hz [203, 204]. Furthermore, preclinical studies and clinical trials have demonstrated that dopamine contributes to the pathophysiology of depression and the preservation of cognitive function and dendritic spine structure [205–207]. Intriguingly, rTMS was found to increase dopamine release in the mesostriatal, mesolimbic, and striatal regions [154, 208].

Furthermore, gamma-aminobutyric acid (GABA) is the primary inhibitory neurotransmitter in the brain [209]. The dysfunction of the GABAergic system contributes to the pathophysiology of depression and cognitive impairment in humans [210, 211]. Significant reductions in GABA levels have been found in severe AD cases, which are involved in the psychological symptoms of AD [210]. Notably, 10-Hz rTMS led to elevated prefrontal cortex GABA in patients with major depressive disorder. In contrast, high-frequency (25 Hz) rTMS could reverse the increased GABA in PFC during aging [212, 213], suggesting rTMS can regulate GABA to a different level in different conditions and maintain the homeostasis of GABA level [212, 213].

#### Attenuates neuroinflammation and regulates glial cells

The anti-inflammatory effects of rTMS have been widely reported in various brain diseases, including Parkinson’s disease [214], focal cerebellar injury [215], ischemic stroke [216], depression [217], and anxiety [218]. Notably, the anti-inflammatory effects of rTMS also contribute to its beneficial role in AD [219]. rTMS at 20 Hz suppressed the overactivation of microglia and pro-inflammatory cytokine levels at the early stage of 5xFAD mice [219]. In AD, the increased release of pro-inflammatory cytokines, such as TNF-α, binds to the cell membrane receptors and induces the intracellular PI3K/Akt/NF-κB signaling pathway activation [219]. The increased NF-κB binds with DNA to further cause the release of pro-inflammatory in both the cortex and hippocampus of AD [219]. However, treatment with rTMS at 20 Hz significantly reduces the phosphorylation of Akt and the translocation of the p65 subunit of NF-κB into the nucleus, suggesting rTMS suppressed the neuroinflammation by inhibiting the excessive activation of PI3K/Akt/NF-κB signaling pathway [219]. Finally, the anti-inflammatory of 20-Hz rTMS significantly improves the neuronal environment and alleviates synaptic plasticity impairment in AD animals [219].

Although there is no direct evidence demonstrating the switch between pro-inflammatory M1 microglia and anti-inflammatory M2 microglia in AD after rTMS treatment, the transformation between M1 and M2 was detected in other brain disease models [10]. In our previous study, rTMS with a standard theta-burst stimulation paradigm (3 pulses of 50 Hz, repeated every 200 ms) were applied to an ischemic stroke model [10]. We found that rTMS effectively induce a switch between M1 and M2 phenotype [10]. Interestingly, a shift in astrocytic A1/A2 phenotype was also detected in this stroke model after rTMS therapy [10]. The release of pro-inflammatory cytokines characterizes M1 and A1 phenotypes, and M2 and A2 are associated with increased production of anti-inflammatory cytokines, the removal of cellular debris, and tissue repair [8]. Intriguingly, consistent with the polarization of glial cells, anti-inflammatory cytokines are increased, and the pro-inflammatory cytokines are decreased [8]. Additionally, the transformation of astrocyte from neurotoxic A1 phenotype to neuroprotective A2 phenotype was also found in ischemic stroke treated with rTMS stimulation at 10 Hz [216].

Moreover, the anti-inflammatory effect of rTMS was also confirmed by in vitro studies [215, 220]. In the primary cortical astrocyte culture, low-frequency and high-frequency stimulation alleviated oxygen-glucose deprivation (OGD) induced-neurotoxic A1 polarization [216]. Similarly, the pro-inflammatory mediator/anti-inflammatory cytokine concentration was also decreased/increased, consistent with in vivo study [216]. Aberrant increased calcium signaling contributes to excessive astrocytic activation and is a good indicator of disease severity/state in neurological disorders [220, 221]. Interestingly, rTMS leads to a significant down-regulation of calcium signal-related genes and inflammatory molecules, suggesting that rTMS has a potential role in regulating the AD inflammatory response [220].

#### Inhibits oxidative stress and preserves mitochondrial function

A large body of evidence demonstrates that oxidative stress is a prominent early event in AD and a promising biomarker for predicting treatment effects [222]. Interestingly, rTMS display its anti-oxidative effects in multiple brain diseases [223, 224]. For example, multiple sclerosis patients who received long-term treatment with 1-Hz rTMS showed significant improvement in various levels of psychometric evaluation and blood analysis [225]. Notably, there is a marked reduction of oxidative stress in the plasma [225]. Moreover, as an approved first-line treatment for depression in many countries [226, 227], rTMS also show its anti-oxidative effect on depression [228]. Although the relationship between reduced oxidative stress and improvement in depression was not established, patients with medication-resistant major depression displayed a significantly decreased level of oxidative stress marker in the blood sample after rTMS treatment [228].

Additionally, effective rTMS at a high frequency can alleviate oxidative stress [229]. In a 3-nitropropionic acid-induced oxidative stress model, the stress rats receiving 60-Hz rTMS displayed significantly decreased oxidative products and improved anti-oxidative enzyme activity in cortical synaptosomes [229]. Interestingly, rTMS did not affect the levels of these oxidative products and enzyme activity in the normal cortical synaptosomes, suggesting that rTMS is only involved in maintaining cellular redox homeostasis in pathological conditions [229]. Similarly, after ischemic stroke, rTMS treatment at 50 Hz significantly suppressed NADPH oxidase activation and superoxide levels in the peri-infarct cortical proteins [224]. It was also accompanied by markedly reduced expression of oxidative neuronal damage markers, including MDA lipid peroxidation marker (MDA) and DNA damage markers (e.g., p-H2A.X Ser139 and 8-OHdG) [224].

The oxidative damage is closely associated with mitochondrial function [22], and the disruption of the mitochondrial membrane was found in various brain diseases [22]. Intriguingly, the disrupted mitochondrial membrane potential is alleviated by rTMS at 50 Hz in ischemic stroke, suggesting the potential role of rTMS in modulating mitochondrial function [10]. Although evidence suggests the beneficial role of rTMS in multiple diseases through its anti-oxidative stress, studies in AD are still rare. In a clinical study, AD patients receiving 20-Hz rTMS targeting the left parietal region showed a significant reduction in blood oxidative damage markers in AD patients [223]. Consistent with the changes in oxidative stress, the cognitive function and the network connection between the hippocampus and the left parietal lobe were significantly improved, suggesting that the antioxidant effects of rTMS contribute to the beneficial effects of rTMS in the treatment of AD [223].

#### Alleviates neuronal apoptosis and provides neuroprotection

Due to the improvement of the above-mentioned multiple factors, rTMS has powerful anti-apoptotic and neuroprotective effects [230]. The Nissl bodies of the neurons in depressed rats were significantly reduced, and the cells were atrophied considerably or degenerated with irregular morphology, suggesting neuronal damage or apoptosis in depressed rats [230]. In contrast, more extensive and granular Nissl bodies were detected in the hippocampal neurons with well-defined cell boundaries after rTMS treatment, suggesting the anti-apoptotic and neuroprotective effect of rTMS in the depression [230]. Furthermore, the expression of the apoptosis regulator BAX was significantly suppressed by rTMS, which further confirmed the anti-apoptotic effect of rTMS in depression [230]. The anti-apoptotic and neuroprotective effect of rTMS is also detected in other disease models with higher rTMS frequency [224]. rTMS treatment at 50 Hz reduced the neuronal apoptosis in the ischemic stroke, supported by significantly suppressing the intrinsic apoptotic pathway in the peri-infarct area [224].

In AD mice, Aβ injection increased neuronal apoptosis, cleaved caspase-3, and Bax levels and decreased levels of Bcl-2 [231]. In contrast, after the treatments with rTMS at 1 Hz, the neuronal apoptosis was significantly suppressed, and apoptosis-related protein expressions were reversed, suggesting the anti-apoptosis effects of rTMS in AD [231]. Furthermore, besides the anti-apoptotic effects, rTMS enhanced the production of the brain-derived neurotrophic factor and nerve growth factor in the brain tissue of AD mice [231]. Both neurotrophic factors are critical molecules in plastic changes associated with learning and memory [231]. These findings suggest that rTMS exerts neurogenic and neuroprotective effects [177, 231].

### Challenges of rTMS therapy in AD

Although rTMS has emerged as a promising approach to slowing down the AD progression or treatment, the evidence regarding long-term efficacy and exact underlying mechanisms is still limited [153]. Additionally, although, as noted earlier, several studies reported positive rTMS effects in AD [179, 232], more advanced clinical trials are still needed to find the therapeutic window for AD treatment [153]. For example, similar to other interventions [44], rTMS might be more effective at the early stage of the disease. Therefore, the rTMS should be applied before the neuronal loss and extensive Aβ deposition in AD [153]. Furthermore, most of the rTMS protocols used to treat AD are similar to those applied in the treatment of medication-resistant depression [153]. There is no agreement on the parameters and protocols of rTMS therapy at which a medication appears to be effective in the clinical application of AD [153]. Moreover, although studies have found that rTMS could correct or blunt the impaired LTP-like plasticity and exert a neuroprotective effect, there is no widely accepted target for rTMS therapy [153, 233]. Furthermore, the modulation of rTMS on mitochondria and glial cells’ transformation has been detected in other brain injuries, but no evidence is found in AD [10]. Finally, although rare (<1% overall), seizures are a potential side effect of TMS treatment [234]. Therefore, the safety of rTMS treatment, how to alleviate this side effect, and determining if there are other possible adverse events of TMS treatment need to be clarified [234].

### Transcranial direct current stimulation (tDCS) and parameters of intervention

Transcranial direct current stimulation (tDCS) is a painless, non-invasive brain stimulation therapy that uses direct current to stimulate specific parts of the brain and produce facilitatory or inhibitory effects [235]. tDCS is typically applied using a constant, low-level current that passes through two electrodes positioned on the scalp to modulate neuronal activity [235]. The tDCS can be divided into two types of stimulation: the positive anodal current stimulation and the negative cathodal current stimulation [235]. The positive anodal current excites neuronal activity in the cortical region under the target electrode, whereas the negative cathodal current reduces or inhibits neuronal activity [235]. As a user-friendly, relatively cheap, and tolerable device, the beneficial effects of tDCS have been studied in various brain diseases, including depression [236], anxiety [237], Parkinson’s disease [238], traumatic brain injury [239], and AD [240]. Although tDCS is not an FDA-approved therapy for AD currently, increasing evidence supports the potential use of tDCS in AD prevention or treatment [241, 242].

### tDCS therapy for AD

#### Improves cognitive function and reduces the deposition of Aβ

In recent years, emerging studies support the promising effect of tDCS in improving cognitive function in AD animals and patients [240, 243]. Repetitive anodal tDCS exhibited distinct neuroprotective effects on the AD brain [243]. To go into greater detail, after 10-day tDCS therapy, the animal showed significantly improved cognitive function and displayed a long-term after-effect that persisted for 2 months [243]. The effects of tDCS on cognitive function have also been investigated in healthy individuals and AD patients [244]. For example, the anodal tDCS could regulate cortical excitability by enhancing depolarization, whereas cathodal tDCS reduces cortical excitability by promoting neuronal hyperpolarization [244]. In human studies, anodal tDCS is applied to stimulate different cortical areas [245, 246]. The anodal tDCS placed on the left prefrontal cortex was able to improve the working memory performance of the healthy individuals [246], which was confirmed by another study in which the tDCS was placed over the left dorsolateral prefrontal cortex (DLPFC) [247]. Interestingly, when the tDCS was placed over the left DLPFC, the improved working memory performance could last for around 30 min, suggesting the potential use of tDCS in individuals with cognitive deficits [247].

In AD patients, anodal tDCS improve visual recognition memory [248], word recognition memory [249], face-name association memory [249], and cognitive function [242]. In a clinical experiment, both the short-term (10 days) and long-term tDCS (10 days/month for 8 months) were applied to AD patients [250]. Results showed that both the short-term and long-term anodal tDCS intervention effectively slowed down the progression of AD and preserved the neuropsychological performance [250]. This clinical experiment also suggests that if an anodal tDCS intervention is effective in the short term by maintaining the neurocognitive function, this beneficial effect can be prolonged over 8 months [250].

Furthermore, tDCS can reduce the formation of amyloid plaques [251]. For example, in an early-stage AD mouse model, 10-day tDCS was applied to the skull over the frontal cortex of the mice [251]. The mice that received tDCS had significantly improved spatial learning and memory, although no improvement in the recognition memory was found [251]. Notably, the Aβ42 deposition was significantly reduced in the tDCS-treated group, which provided direct evidence for alleviating the specific pathological change of AD tDCS [251]. In preclinical AD, the tDCS exhibited its beneficial role in alleviating neurovascular unit dysfunction and reducing Aβ plaques, indicating that the effect of tDCS on Aβ deposition may partly depend on BBB-mediated clearance of Aβ [252].

#### Improves cerebral blood flow

Previous studies found brain hypoperfusion or decreased cerebral blood flow in individuals with mild to moderate AD [253, 254]. Moreover, the compromised cerebral blood flow has been recognized as one of the early events of AD, predicting the progression of the disease and closely correlating with cognitive decline [255, 256]. Improving cerebral blood flow could alleviate the cognitive deficits in the AD animal model and human patients [257–259]. Intriguingly, tDCS show its ability to improve cerebral blood flow in healthy individuals during stimulation [260]. However, the cerebral blood flow returned to baseline when the tDCS was removed [260]. Moreover, cerebral blood flow change exhibited a linear relationship with tDCS intensity [260], suggesting that the regulation of tDCS on cerebral blood flow is intensity-dependent.

Unlike anodal tDCS, the cathodal tDCS induced a reduced cerebral blood flow during stimulation and a continuous decrease compared to baseline [260]. Similar to the human finding, the anodal tDCS also increased cerebral blood flow in rats [261]. However, unlike the human study noted previously, the effect of tDCS can last after tDCS therapy, suggesting that tDCS may have long-lasting after-effects in regulating cerebral blood flow [261]. Consistent with this result, there is an increased oxygen delivery in the vicinity of the anode after tDCS with after-effects lasting for several minutes after stimulation [262].

#### Alleviates impaired synaptic plasticity

Synaptic plasticity is the ability of synapses to change their strength or efficacy of synaptic transmission in response to synaptic activity changes [263]. In AD, impaired synaptic plasticity is an early event involved in the cognitive deficits [264]. Targeting the impaired synaptic plasticity with the brain stimulation techniques has been considered a powerful approach for treating AD [265]. Interestingly, the tDCS is one such method [266]. tDCS could induce membrane polarization and polarity-specific shifts of cortical excitability during and after stimulation and modulate the conductance of sodium and calcium channels [266]. These changes are involved in neuroplastic changes and provide a possible mechanism underlying long-lasting after-effects [266].

Moreover, the long-lasting after-effects of tDCS depended on NMDA receptor-associated neuroplastic changes [266]. The changes in NMDA receptor and intracellular Ca^2+^ uptake are one of the primary physiological bases of tDCS [267]. As mentioned previously, anodal tDCS enhances cortical activity, while cathodal stimulation reduces cortical excitability [244]. When the NMDA receptor was activated by anodal tDCS, the intracellular Ca^2+^ within the postsynaptic neuron was significantly increased [267, 268]. The effect of tDCS on synaptic plasticity depends on the degree of NMDA receptor activation [267, 268]. A slight increase of the intracellular Ca^2+^ in the postsynaptic neuron will lead to LTD-like changes [267, 268]. On the other hand, a moderate rise in Ca^2+^ does not affect the synaptic plasticity, and a more significant increase induces LTP-like changes [267, 268].

#### Regulates neurotransmitter systems

As mentioned previously, the AD rat model improved long-lasting cognitive function and memory performance after tDCS therapy [243]. The molecular study found decreased choline acetyltransferase (ChAT) levels in AD rats. In contrast, a notable increase of ChAT in the tDCS group [243] suggests that neurotransmitters’ changes may be involved in the effects of tDCS. ChAT is a transferase enzyme that facilitates the synthesis of the neurotransmitter acetylcholine (ACh) [269]. It is assumed that ChAT plays a crucial role in learning and memory [269]. In the AD rat model, Aβ-induced neurotoxicity led to the cholinergic system’s disruption and decreased ACh level [270]. Therefore, the increased expression of ChAT after tDCS therapy may improve ACh’s concentration and exerts benefits in AD. Furthermore, this effect even lasts 2 months after tDCS [243]. In addition to its impact on ChAT and ACh, tDCS can also affect other neurotransmitters [271]. In healthy individuals, the effect of tDCS is mediated by multiple monoamine neurotransmitters (e.g., dopamine and serotonin) [272–274], and the regulation of these neurotransmitters may contribute to the neuroprotective role of tDCS in AD.

#### Ameliorates neuroinflammation

tDCS also displays pronounced anti-inflammatory effects in AD. In AD, the deposition of Aβ oligomers induces astrocyte activation [9, 44]. The excessive activation of astrocytes will release a large number of pro-inflammatory, which in turn facilitate the deposition of amyloid plaques [9, 44]. In an AD mouse model, tDCS reduces astrocyte activation in different brain areas, including CA1-CA3 and dentate gyrus [251]. Not only in AD, but the anti-inflammatory effects of tDCS have also been found in other dementia [275]. For example, in a rat model of vascular dementia, anodal tDCS significantly suppressed the over-activation of microglia and astrocyte, accompanied by attenuation of demyelination in both the corpus callosum and internal capsule [275]. Consistent with the changes in glial cells, the increased levels of pro-inflammatory factors (e.g., IL-1β, IL-6, and TNF-α) were alleviated by anodal tDCS [275]. Interestingly, cathodal and anodal tDCS promote microglial activation in the healthy brain [276]. However, in an ischemic stroke, the effect of tDCS on microglia is different from that in the healthy brain [277]. A study showed that cathodal tDCS could reduce the excessive activation of microglial and alleviate inflammation in the peri-ischemic cortex [277]. Therefore, the effects of tDCS may also depend on the health conditions of the brain.

### Challenges of tDCS therapy in AD

Similar to PBM therapy and rTMS, the effects of tDCS depend on the intensity, the duration, the position of the electrode over the scalp, the number of sessions of tDCS, and the mode of stimulation [235]. Although a more significant number of clinical trials have been performed to measure the effects of tDCS on AD, there are no standard protocols regarding the clinical use of tDCS [278].

Another question is how to extend the after-effect. As mentioned previously, the after-effect of the tDCS usually only lasts several minutes [247]. Studies that focus on finding the optimal parameters to extend the after-effect of the tDCS may help improve the beneficial effect of tDCS.

Third, the effects of cathodal and anodal tDCS in the different animal models are inconsistent [275, 277]. Although, as mentioned previously, most studies only found beneficial effects of anodal tDCS [245, 246]. Inconsistences still exist. For example, a previous study found that cathodal stimulation could protect cortical neurons against ischemic damage and facilitate clinical recovery [277]. However, anodal tDCS deteriorated postischemic lesion volume and augmented the derangement of the blood-brain barrier [277]. Therefore, more studies are still needed to clarify the effect of anodal tDCS and cathodal tDCS.

Finally, more studies are required to investigate the specific target of tDCS. For example, there is a very close association between light, electricity, and magnetism [279]. Mitochondria is a primary target of PBM and a mediator of rTMS, but studies investigating the effect of tDCS on mitochondria in AD are less numerous. However, other neurodegenerative diseases provided insights into the mitochondrial changes with tDCS therapy in AD [280]. For example, in a Parkinson’s disease (PD) mouse model, the PD animal showed pronounced mitochondrial damage, as evidenced by mitochondrial swelling, decreased mitochondrial glutamate dehydrogenase, and decreased ATP content in the substantianigra [280]. In addition, the PD mouse model also displayed reduced mitochondrial biogenesis-related protein and significantly elevated fission-associated protein (Drp1), indicating mitochondrial biogenesis deficits and excessive mitochondrial fragmentation [280]. Interestingly, these changes were alleviated by anodal tDCS, which implies a potential role of tDCS in protecting mitochondria [280]. However, to the best of our knowledge, we did not find research investigating whether mitochondria are one of the potential targets of tDCS.

### Exercise and parameters of intervention

Substantial evidence indicates that physical excise is a known modifiable risk factor for AD [281]. In addition, physical exercise slows down AD progression and development and helps combat other AD risk factors and complications [9, 44, 282]. Many studies demonstrate the neuroprotective of exercise interventions [9, 44, 281, 282]. Compared with other pharmacological and other non-invasive therapies, physical exercise is easily accessible and affordable [281]. The effects of exercise can be affected by four elements, including intensity, time, frequency, and type of exercise [283]. Physical activity can be categorized into low, moderate, and vigorous intensity measured according to the heart rate or percentage of the maximal oxygen consumption (can be measured by the metabolic equivalent of task) [284]. In AD, the type of exercise intervention is also an essential factor affecting exercise benefits [285]. The types of exercise can currently be classified into four categories: strength, flexibility, endurance, and balance. In AD studies, aerobic exercise is the most well-studied endurance exercise and displays beneficial effects [9, 44]. According to previous studies, exercise interventions can improve cognitive function, improve neuroplasticity, and reduce neuropsychiatric symptoms [9, 44]. Similar to other non-invasive theories, exercise also benefits AD treatment or prevention [9, 44].

### Exercise therapy for AD

#### Alleviates learning and memory deficits and anxious-depressive-like behaviors

A large body of studies has provided evidence supporting the beneficial effects of physical excise in preserving learning and memory function [9, 44]. In multiple animal models of AD, physical exercise has worked as a pre-/post-treatment strategy in improving cognitive function or preventing memory loss [9, 44]. For example, in the STZ-induced sporadic AD rat model, animals for 4-week post-intervention using treadmill exercise displayed significantly improved hippocampal-dependent cognitive functioning, including the improved spatial learning and memory and recognition memory [286]. Similarly, in a transgenic AD mouse model with Tau pathology, after 9 months of exercise, AD-like mice from the long-term voluntary exercise groups showed significant improvement in short-term working memory [287]. Moreover, even shorter voluntary activity for 5 months also confers a beneficial role in improving learning and memory [287]. In APP/PS1 mouse model, 12-week treadmill exercise significantly improved cognitive function, suggesting the improvement of cognitive function with physical exercise can be found in different types of physical activity with different exercise intensity or duration [288]. In addition to exercise post-treatment, increasing evidence demonstrated the preventive effect of exercise on cognitive deficits [44]. For example, 4-week swimming exercise pretreatment significantly attenuated STZ-induced learning and memory deficits, including hippocampus-dependent spatial learning and memory and recognition memory [44]. Furthermore, consistent with the improvements in cognitive function, the expressions of synaptic proteins were well preserved in the exercise pretreatment group [44].

According to previous studies, anxious-depressive-like behavior worked as both a predictor and a causal factor contributing to AD progression [9, 289]. In clinical studies, AD patients had significantly increased anxious-depressive symptoms, and individuals with psychological distress or anxiety are more likely to be diagnosed with AD [289–292]. Moreover, anxious-depressive behaviors are closely related to extensive amyloid plaques and neurofibrillary tangles [9, 290, 293]. Interestingly, exercise pretreatment attenuated anxious-depressive-like behavior and reduced typical pathological changes at the early stages of AD [192]. However, cognitive deficits did not occur at that time, suggesting physical exercise is a practical approach to prevent early-stage behavioral changes in the transgenic AD rat model [192]. Similarly, 6 months of voluntary physical training also shows its beneficial effect in ameliorating anxiety levels in a transgenic AD mouse model. Consistent with the results of animal studies, clinical trials show that AD patients with depression displayed a clear benefit after participating in the physical exercise [294]. However, more studies with a larger sample are still needed to clarify the benefits of different types of exercise in AD [294].

#### Increases cerebral blood flow

According to a previous study, AD manifests as a significant decrease in cerebral blood flow (CBF) [295]. Compared to age-matched healthy controls, the total blood flow of AD patients was reduced by 40% [296]. Even patients with mild cognitive impairment (MCI) have a significant decrease in CBF [297]. In AD, several specific regions are more likely to show CBF decreases, including the hippocampus, a critical area for memory [296–298]. CBF is closely related to the severity of AD patients and is a possible target in healthy elderly or MCI patients [297, 299]. Previous findings have suggested the association between Aβ accumulation and CBF among elderly adults across the span from controls to AD patients [300]. The higher Aβ deposition is associated with more significant CBF reduction and worse memory [300]. The increase of cerebral blood flow during exercise has been well documented in healthy individuals [301, 302], although a few studies showed a decrease or no change in CBF during physical exercise [303, 304]. Multiple factors led to the increase of CBF during physical activity [305], including the changes in arterial partial pressures of carbon dioxide (pCO2), arterial pressure, cardiac output, muscle mechanoreceptors, oxidative metabolism, and neural innervation [305, 306]. Muscle mechanoreceptors contribute to the increase of CBF at the initial exercise stage, indicating physical exercise’s beneficial role in attenuating AD pathological changes [307]. The pCO2 regulates CBF through pH changes by modulating the cerebral vessel diameter after initiating physical activity [308]. In the human study, increased CBF was found in AD patients after 12 months of aerobic exercise training [309], suggesting that the increased CBF after exercise may contribute to the effects of physical activity on the brain.

#### Inhibits the release of inflammatory factors and gliosis

As mentioned previously, the increased/decreased release of pro-inflammatory/anti-inflammatory factors and the changes in glial cell phenotype are implicated in the pathogenesis and development of AD [9, 22, 310]. In the STZ-induced rat model of sporadic AD, the pro-inflammatory factors (e.g., IL-1β, TNFα) are significantly increased, and the anti-inflammatory factors (e.g., Il-4, IL-10) are decreased in the hippocampal CA1 region [286]. However, 4-week treadmill exercise post-treatment alleviated these changes and promoted microglial polarization from M1 pro-inflammatory phenotype to M2 anti-inflammatory phenotype [286]. Similarly, in the STZ-induced rat model of sporadic AD, exercise pretreatment also displayed its anti-inflammatory effects [44]. In one previous study, 4 weeks of treadmill training pretreatment significantly ameliorated reactive gliosis and neuroinflammation [44]. Although the switch between M1 and M2 phenotype was not mentioned, the increased release of anti-inflammatory cytokines and decreased pro-inflammatory cytokines were found in the exercise pretreated group [44]. In addition to regulating the microglial phenotype, physical exercise also modulates the astrocytic phenotypes [311]. In the brain of AD patients, neurotoxic phenotype A1 was predominant [61, 312]. Interestingly, exercise promotes the transformation of astrocytes from neurotoxic A1 phenotype to neuroprotective A2 phenotype [311].

In most AD animal studies, exercise suppresses the overactivation of glial cells, including astrocyte and microglia, which is consistent with inflammatory factors [9, 44]. However, the study also found increased astrocytes in the hippocampus after long-term voluntary physical exercise [313]. Interestingly, the beneficial effects of physical training on gliosis are supported by reduced A1 markers and increased A2 markers [313]. Consistent with astrocyte changes, astrocyte-derived BDNF was significantly increased, suggesting astrocyte remodeling after exercise is implicated in the neuroprotective effect of physical activity [313].

#### Promotes oxidative stress-related adaptations and alleviates oxidative damage

Increasing evidence strongly supports a general anti-oxidative role of exercise in AD [44]. In an aged AD-like mouse model, analysis of brain tissue showed high levels of oxidative damage [314]. The excessive produced ROS reacts with protein, lipids, and nucleic acids to generate protein carbonyls, lipid peroxides, and nucleic acid oxidative modification [44]. However, the oxidative damage was significantly ameliorated by exercise by maintaining redox homeostasis [314]. Furthermore, network analysis of oxidative stress markers and AD-like pathologies showed that oxidative stress might be a major event and a primary target for exercise neuroprotection against AD pathological changes [314]. In the STZ-induced sporadic AD animal model, 4-week treadmill exercise led to a robust inhibition of oxidative damage to protein, lipids, and nucleic acids, which contributes to the neuroprotection and cognitive improvement after exercise post-treatment [286]. Moreover, exercise pretreatment also has an anti-oxidative effect in the sporadic AD animal model [44]. Similar to exercise post-treatment, swimming exercise pretreatment significantly attenuated oxidative damage to proteins and lipids [44]. In addition, exercise preserved total anti-oxidative capacity and improved the expression of antioxidant enzymes, including superoxide dismutase 2 (SOD2), thioredoxin 2 (TRX2), heme oxygenase 1 (HO-1), and NAD(P)H dehydrogenase [quinone] (NQO1) [44]. Further analysis found increased nuclear erythroid 2-related factor 2 (Nrf2) DNA-binding activity in the exercise pretreatment group [44]. Nrf2 is a critical molecule in regulating the cellular antioxidant ability [315]. In AD, the protective role of Nrf2 is compromised [44, 315]. However, exercise pretreatment promotes the translocation of Nrf2 from the cytoplasm into the nucleus and binds to the antioxidant response element (ARE) to active expressions of antioxidant enzymes, which is consistent with the changes of SOD2, TRX2, HO-1, and NAD(P)H dehydrogenase [quinone] (NQO1) after exercise treatment [44]. The anti-oxidative effect of exercise is also found in numerous human clinical trials [316, 317]. For example, a previous study found reduced ROS levels, increased antioxidant capacity, and decreased oxidative damage by-products in the blood or urine samples after 6 weeks of physical exercise [316, 317].

#### Improves glymphatic clearance

The glymphatic system is a glial-dependent waste clearance system that utilizes an astroglia-formed system of the perivascular channels to remove the toxins and metabolic waste from the brain in the central nervous system (CNS) of vertebrates [318]. The brain’s glymphatic system’s disruption or compromised function is implicated in the deposition of misfolded proteins in aging and neurodegenerative disease [318]. In the glymphatic system, the paravascular cerebrospinal fluid (CSF)-interstitial fluid (ISF) exchange plays a central role in the clearance of interstitial solutes [319]. The exchange of ISF and CSF along perivascular spaces depends on Aquaporin 4 (AQP4), a protein located in the end-feet of the astrocyte, which is the major component of the glymphatic system [320]. The transgenic mice lacking AQP-4 exhibit significantly declined interstitial solute clearance compared with wild animals [319]. Under physiological conditions, the CSF flows across highly polarized AQP4 molecules at astrocyte end-feet, mixing with ISF, which allows the clearance of Aβ [321, 322]. However, under pathological conditions in AD, both the AQP4 expression and polarization are reduced, and AQP4 is redistributed from the end-feet of the astrocyte to the cell body, which induces impaired ISF drainage and compromised glymphatic clearance [321–323]. In the APP/PS1 AD mouse model, AQP4 knockout exacerbated AD mice’s cognitive deficits and other typical pathological changes [324]. Interestingly, in aged mice with cognitive deficits, 6-week voluntary exercise promotes glymphatic clearance of Aβ by regulating AQP4 polarization and improving the expression of AQP4 in the perivascular regions [322]. In addition, the increased heart rate, perfusion flow, cerebral pulse pressure, and brain interstitial fluid flow after exercise also suggest that the improvement of glymphatic clearance mediates the neuroprotective effects of exercise [319, 325]. Moreover, although a previous study found that voluntary wheel running could not affect BBB permeation, the glymphatic clearance of Aβ and the BBB disruption have been improved following physical exercise, indicating exercise may promote BBB-mediated clearance of Aβ or tau [322].

#### Preserves mitochondrial bioenergetics, mitochondrial biogenesis, and quality control

Mitochondrial adaptations are another crucial underlying mechanism for exercise-induced neuroprotection [326]. Exercise-induced mitochondrial adaptations include preserved mitochondrial bioenergetics, mitochondrial biogenesis, and mitochondrial quality control [326]. The decreased activity of enzymes in the mitochondrial electron transport chain (ETC), dysregulation of calcium homeostasis, reduced antioxidant enzymatic activity, and increased ROS generation led to mitochondria dysfunction and, therefore, decreased ATP synthesis [22, 44, 327]. However, in the brain of aged mice, a total of 17 days of treadmill training significantly improved the activity of enzymes in ETC and increased rates of mitochondrial respiration and ROS production [328]. Notably, the increased ROS generation after exercise differs from the pathological ROS accumulation [329]. Exercise-induced ROS generation is a moderate and short-term increase, which induces mitochondrial adaptions to oxidative stress and improves the brain’s enzymatic antioxidant system [329]. Intriguingly, in the transgenic mouse AD model, the pathological increased hippocampal ROS accumulation in the AD mouse model was reduced after long-term treadmill running [330]. In the same AD animal model, the impaired mitochondrial function was restored by 20-week treadmill running, as evidenced by increased mitochondrial state 3 and 4 respiration [330]. Furthermore, the impaired complexes I, IV, and ATP synthase activities were improved in the hippocampus of AD mice with exercise training [330]. This evidence demonstrates that exercise training preserves mitochondrial bioenergetics and exerts neuroprotection in AD [328, 330].

Mitochondrial biogenesis is the guarantee for the number of mitochondria and the energy requirement in neurons [331]. However, mitochondrial biogenesis is impaired in AD by increased mtDNA mutations and decreased peroxisome proliferator-activated receptor gamma coactivator 1-alpha (PGC-1α) [332]. In addition, mitochondrial biogenesis requires the coordinated synthesis of proteins encoded by mtDNA and nuclear DNA [331]. However, the increased mtDNA mutations and decreased expression of PGC-1α and its downstream target have been widely reported in AD [22, 331]. Intriguingly, 6-month exercise training alleviates the damaged mtDNA in the transgenic AD mouse model [333]. Similarly, in the APP/PS1 transgenic mouse model, 20-week treadmill training improves mtDNA repairability in the mouse hippocampus, which protects against the impairment of mitochondrial function and mitochondrial biogenesis [330].

As mentioned previously, the balance between mitochondrial fission and fusion is crucial for maintaining a healthy mitochondrial pool and is essential for cellular function and stress responses [28]. AD disrupts this balance, causing excessive mitochondrial fragmentation and impaired mitochondrial function [22]. However, evidence suggests that exercise regulates mitochondrial dynamics and maintains mitochondrial homeostasis [331, 334]. A previous study found that under physiological conditions, the phosphorylation of mitochondrial fission-related protein Drp1 was increased immediately after acute resistance exercise, suggesting the acute exercise triggers the self-protective mechanism against mitochondrial damage by segregating the damaged mitochondria [335]. In contrast, long-term resistance training improves mitochondrial fusion-related protein to prevent excessive mitochondrial fission and maintain mitochondrial function [335]. In APP/PS1 transgenic mice, moderate-intensity continuous training and high-intensity interval training down-regulated the expression of the mitochondrial fission-related protein (e.g., Drp1 and Fis1). In contrast, the expression of mitochondrial fusion-related proteins (e.g., MFN1, MFN2, and OPA1) was improved, accompanied by alleviation of cognitive dysfunction, enhanced the activity of antioxidant enzymes, and decreased oxidative stress [336].

Interestingly, improved mitochondrial quality control after exercise training was found in other aging-related diseases, including sporadic inclusion body myositis and Parkinson’s disease [337, 338]. Furthermore, in a human study, both the mitochondrial fission and fusion-related proteins were increased in the active subjects compared with the respective sedentary ones, suggesting exercise promotes the efficiency of mitochondrial quality control by improving the expressions of mitochondrial dynamics associated proteins [339]. Moreover, research on an AD mouse model found that the efficiency of exercise therapy on mitochondrial dynamics was regulated by PGC1a/FNDC5/irisin pathway, a sensitive pathway in response to physical exercise, suggesting the potential role of exercise on mitochondrial dynamics in AD [340].

### Challenges of exercise therapy in AD

Similar to other non-invasive approaches, questions still focused on which intensity is the optimal dosage? Which is the best exercise form for preventing or slowing down AD? Furthermore, another question that remains unanswered is investigating the optimal timing of initiation of physical exercise treatment. The earlier the exercise treatment begins, the better the outcomes, or does the individual enjoy a similar beneficial effect even to exercise after mid-life or later life? Additionally, although numerous studies support the beneficial role of exercise in AD, a study found that exercise appears not to affect working memory deficit [341]. Therefore, more studies are still needed to clarify the effects of physical activity on different types of memory tasks. To date, although significant progress has been achieved in animal studies, no randomized controlled trials demonstrate that exercise can prevent dementia [9, 44, 282, 342]. For example, according to a previous study, there are no noticeable improvements in cognitive function in people with mild to moderate dementia [342]. Therefore, more studies on humans are needed to determine why the effects of exercise are so variable. Additionally, a previous study found that 6 months of exercise intervention had gender differences in cognition. The women performed better on cognitive function-based tasks than men [343]. Therefore, gender difference is a major factor that must be considered in AD studies. Finally, more animal and human studies are still required to elucidate the underlying molecular mechanism of physical activity in AD [310]. For instance, the BBB breakdown has been confirmed in both the AD animal and postmortem AD patients [344]. However, to the best of our knowledge, we did not find enough evidence to demonstrate the effectiveness of exercise on BBB.

### Summary of effects, mechanism, and future directions

**Fig. 7:**
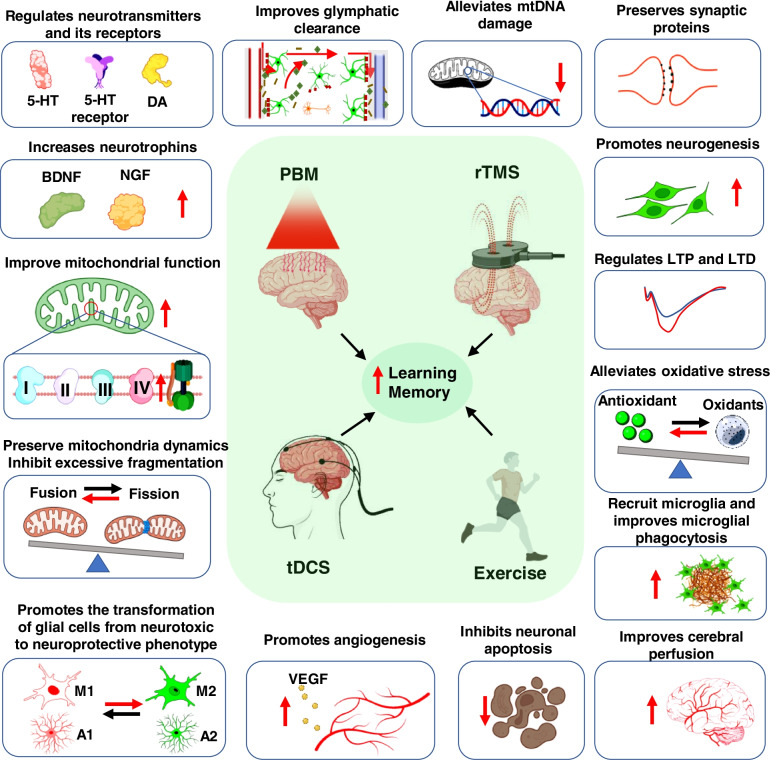
Effects and mechanisms for non-invasive therapies in AD. These non-invasive treatments are able to alleviate neuronal apoptosis, improve cerebral perfusion, recruit microglia around amyloid plaques, improve microglial phagocytosis, attenuate oxidative stress, regulate LTP and LTD, promote neurogenesis, preserve synaptic proteins, alleviate mtDNA damage, improve glymphatic clearance, regulate neurotransmitters, increase neurotrophins, maintain mitochondrial function and mitochondrial dynamics, promote the transformation of glial cells from neurotoxic to neuroprotective phenotype, and promote angiogenesis

As discussed previously, these non-invasive approaches are promising therapeutic methods for slowing AD progression or alleviating AD typical symptoms [9, 44, 69, 70, 240, 243]. For example, the studies discussed offer compelling evidence that non-invasive approaches, including PBM, rTMS, tDCS, and physical exercise, can alleviate memory loss and reduce pathological hallmarks of AD (e.g., amyloid plaques and tau pathology) [69, 102, 251]. In addition, as shown in Fig. 7, the beneficial effects of these non-invasive treatments include alleviating neuronal apoptosis, improving cerebral perfusion, recruiting microglia around amyloid plaques, improving microglial phagocytosis, attenuating oxidative stress, regulating LTP and LTD, promoting neurogenesis, preserving synaptic proteins, alleviating mtDNA damage, improving glymphatic clearance, regulating neurotransmitters, increasing neurotrophins, maintaining mitochondrial function and mitochondrial dynamics, promoting the transformation of glial cells from neurotoxic to neuroprotective phenotype, and enhancing angiogenesis. Although these factors that contribute to the beneficial effects of non-invasive treatments seem independent, they are interconnected and affect each other. For instance, the improvement of the microenvironment significantly enhances neurogenesis, promotes angiogenesis, and alleviates neuronal apoptosis [10, 121]. Mitochondrial function, mitochondria-associated oxidative stress, and neuroinflammation are central factors in modulating the neuronal microenvironment [22].

The failure of pharmacological therapy targeting only single AD-related pathology suggests that multi-target therapy is a potential approach to AD treatment [345]. Non-invasive therapies discussed in this review target multiple pathological changes in AD and display promising effects in improving cognitive function and anxious-depressive-like behavior. Therefore, to improve the translational value of these non-invasive therapies, it will be critical to understand the underlying mechanisms and find the optimal therapy parameters in clinical application. Moreover, the combined use of non-invasive therapies is also a promising research direction for future studies [346].

## Conclusions

Non-invasive therapies have the potential and promise to achieve therapeutic goals of slowing down or preventing AD. As shown in Table 1, the improvements in cognitive function and other behavioral changes after non-invasive treatments rely on multiple cellular changes and adaptive responses. The challenges to advancing non-invasive treatments in AD prevention and treatment are shown in Table 2. No agreement on the optimal therapy parameters in clinical application and finding the exact or specific targets are the primary challenges for future studies. If future studies are successful in these non-invasive approaches, they will shed light on AD treatment and prevention.

**Table 1:** Effects of non-invasive therapy for Alzheimer’s disease

Therapy	Effects of non-invasive therapy and underlying mechanisms
PBM	• Improves spatial learning and memory [69, 70, 105] • Improves the auditory sentence comprehension [171] • Increases the ability of Aβ phagocytosis [69, 70] • Reduces the levels of Aβ1-40 and Aβ1-42 [69, 70] • Regulates microglia’s morphological transformation [70] • Upregulates VEGF levels to promote angiogenesis [69] • Alleviates the tau hyperphosphorylation [102] • Attenuates anxious-depressive-like behavior [92, 115–117] • Protects against neuronal damage, degeneration, and apoptosis [7] • Improves cerebral perfusion and resting-state functional connectivity [105] • Enhances mitochondrial cytochrome c oxidase (complex IV) activity [8, 22] • Preserves mitochondrial dynamic and inhibits mitochondrial fragmentation [102, 125, 126] • Recruits microglia around amyloid plaques and improves microglial phagocytosis [70] • Promotes the transformation of microglia from a neuroprotective to a neurotoxic phenotype and inhibits neuroinflammation [69, 102] • Inhibits oxidative stress and oxidative damage by activating NF-κB and PI3K/Akt pathway [125, 142–145]
rTMS	• Improves learning and memory [171, 174, 176, 177] • Decreases Aβ accumulation and tauopathy [174, 175, 179] • Improved auditory sentence comprehension [175] • Regulates long-term potentiation/depression (LTP/LTD) by modulating the strength of Ca^2+^ internal flow and the intracellular Ca^2+^ level in the postsynaptic membrane [181] • Promotes neurogenesis and the differentiation of newborn cells into mature neurons [186] • Promotes the expressions of synaptic protein markers [178, 182] • Enhances brain-derived neurotrophic factor (BDNF)/tropomyosin-related kinase B [178, 182] • Inhibits oxidative stress [223] • Regulates neurotransmitters and their receptors (e.g., 5-HT content, 5-HT receptors, dopamine, gamma-aminobutyric acid) [154, 199–202, 208] • Alleviates the impairment of synaptic plasticity [219] • Inhibits neuroinflammation through PI3K/Akt/NF-κB signaling pathway [219] • Exerts neurogenic and neuroprotective effects by improving the production of the brain-derived neurotrophic factors [177, 231]
tDCS	• Improves cognitive function and reduces amyloid plaques [242, 251] • Improves cerebral blood flow [261] • Regulates synaptic plasticity by modulating membrane polarization, cortical excitability, and NMDA receptor [266] • Regulations on neurotransmitter systems [272–274] • Reduces the excessive activation of glial cells and inhibits neuroinflammation [251, 275]
Exercise	• Alleviates learning and memory deficits and anxious-depressive-like behaviors [192, 286] • Increases cerebral blood flow [301, 302, 309] • Inhibits the release of inflammatory factors and gliosis [44, 286] • Promotes the transformation of astrocytes from neurotoxic A1 phenotype to neuroprotective A2 phenotype [311] • Promotes astrocytic brain-derived neurotrophic factor [313] • Promotes oxidative stress-related adaptations and alleviates oxidative damage [44, 286, 314] • Promotes Nrf2 DNA-binding activity [44] • Improves glymphatic clearance [319, 322, 325] • Enhances the activity of enzymes in ETC and the rates of mitochondrial respiration [328] • Induces mitochondrial adaptions to oxidative stress and improves the brain’s enzymatic antioxidant system [329] • Alleviates the damage of mtDNA [330] • Preserves mitochondrial dynamics and maintains mitochondrial homeostasis [331, 334] • Promotes the efficiency of mitochondrial quality control by improving the expressions of mitochondrial dynamics associated proteins [339]

*VEGF* Vascular endothelial growth factor, *Nrf2* Nuclear erythroid 2-related factor 2

**Table 2:** Challenges for non-invasive therapies in AD treatment

Treatments	Challenges for non-invasive therapies
PBM	• No agreement on the parameters of PBM therapy in the clinical application [96] • There are still have conflicting data doubting whether it is the primary acceptor or target of PBM therapy [148, 149] • The precise mechanisms of PBM treatment, especially the exact mechanisms of pulsed-wave PBM therapy in AD, remain elusive [69, 70] • The effects and mechanisms of indirect or remote PBM therapy remain to be understood [150, 151]
rTMS	• Evidence regarding long-term efficacy and exact underlying mechanisms is still limited [153] • More advanced clinical trials are still needed to find the therapeutic window [153] • No agreement on the parameters and protocols of rTMS therapy at which a medication appeared to be effective in the clinical application of AD [153] • More studies on the regulation of mitochondria and glial cells’ transformation are needed • The safety of rTMS treatment needs to be clarified [234]
tDCS	• No standard protocols regarding the clinical use of tDCS [278] • Finds ways to extend the after-effect of the tDCS [247] • The effect of anodal and cathodal tDCS needs to be clarified [234] • More studies are required to investigate the specific target of tDCS
Exercise	• No agreement on the optimal dosages, the best types of exercise, and the optimal timing of initiation of physical exercise for prevention or slowing down AD • The effects of exercise in different types of memory tasks are variable [9, 44, 282, 342] • The gender difference is one of the major factors that need to be considered [343] • More animal and human studies are still required to elucidate the underlying molecular mechanism of physical activity in AD (for instance, the effectiveness of exercise on BBB) [310, 344]

*PBM* Photobiomodulation, *rTMS* Transcranial magnetic stimulation, *tDCS* Transcranial direct current stimulation

## Supplementary Information

**Additional file 1: Supplementary Table 1.** Non-invasive Therapies in Clinical Trials.

## Data Availability

Not applicable.

## Abbreviations

AD: Alzheimer’s disease
PBM: Photobiomodulation
TMS: Transcranial magnetic stimulation
tDCS: Transcranial direct current stimulation
APP: Amyloid precursor protein
NFTs: Neurofibrillary tangles
ATP: Adenosine triphosphate
ROS: Reactive oxygen species
DAMPs: Damage-associated molecular patterns
IL-3: Interleukin-3
PS1: Presenilin-1
LLLT: Low-level laser (light) therapy
CCO: Cytochrome C oxidase
PHF1: Hyperphosphorylated tau
NIR: Near-infrared light
NO: Nitric oxide
4HNE: 4-Hydroxyhexenal
8-OHDG: 8-Hydroxydeoxyguanosine
rTMS: Repetitive transcranial magnetic stimulation
MDD: Major depressive disorder
FDA: Food and Drug Administration
TBS: Theta-burst stimulation
MCI: Mild cognitive impairment
LTP: Long-term potentiation
LTD: Long-term depression
BDNF: Brain-derived neurotrophic factor
TrkB: Tropomyosin-related kinase B
5-HT: Serotonin
DLPFC: Dorsolateral prefrontal cortex
GABA: Gamma-aminobutyric acid
OGD: Oxygen-glucose deprivation
ChAT: Choline acetyltransferase
Ach: Acetylcholine
PD: Parkinson’s disease
CBF: Cerebral blood flow
SOD2: Superoxide dismutase 2
TRX2: Thioredoxin 2 (TRX2)
HO-1: Heme oxygenase 1 (HO-1)
NQO1: NAD(P) H dehydrogenase [quinone]
Nrf2: Nuclear erythroid 2-related factor 2
ARE: Antioxidant response element
CNS: Central nervous system
AQP4: Aquaporin 4
ETC: Electron transport chain
PGC-1α: Proliferator-activated receptor gamma coactivator 1-alpha
BBB: Blood-brain barrier
