# Supplementary material for: Therapeutic non-invasive brain treatments in Alzheimer’s disease: recent advances and challenges
Source: Inflamm Regen. 2022 Oct 3;42:31. doi: 10.1186/s41232-022-00216-8 (PMC9527145; doi:10.1186/s41232-022-00216-8)
Supplement: Supplementary file 1 — Additional file 1: Supplementary Table 1. Non-invasive Therapies in Clinical Trials. [file 41232_2022_216_MOESM1_ESM.docx]

**Supplementary Table 1. Non-invasive Therapies in Clinical Trials**

| **Trials Identifier or PMID** | **Conditions** | **Device or Approach** | **Target Region** | **Non-invasive Interventions** | **Recruitment Status** | **FDA-Status** |
| --- | --- | --- | --- | --- | --- | --- |
| NCT02537626 | AD | Erchonia ALS Laser | Frontal cortex, temporal regions, and base of the skull | 8 times, 2 times each week for 4 weeks, 10 min each time | Completed | N/A |
| NCT04496778 | AD  with mild cognitive impairment | Laser Watch- 650 nm; Nasal Probe with 2 laser beams | Left wrist; nose cavity | Combined with aerobic exercise; 30 minutes per session, 2-times/day, 3 days/week for 3 months | Completed | N/A |
| NCT03672474 | AD | REGEnLIFE RGn530 | Head; abdomen | 8 weeks with 5 sessions of 25 minutes per week, for a total of 40 sessions | Terminated (Covid19 sanitary crisis) | N/A |
| NCT04784416 | AD with mild cognitive impairment | Active tPBM-2.0  (Pulsed, 808nm wavelength laser) | Forehead bilaterally | 10 min per day, 3 days per week, for 8 weeks | Recruiting | Phase 2 |
| NCT03160027 | Dementia, Alzheimer type | Vielight Neuro Gamma (NIR lights at 40 Hz) | Transcranial and intranasal near infrared (NIR) light | Once every other day for 20 minutes for 12 weeks. | Completed | N/A |
| NCT01059877 | Dementia | 1060-1080 nm light emitting diodes | Cortex | 6 minute for 28 days | Completed | N/A |
| NCT04055376 | AD | GENUS device (light and sound stimulation at gamma frequency) | Eye, ear | 1 hour per day for 6 or 9 months | Active, not recruiting | Phase 2 |
| NCT02518412 | AD | Low DCS (1-2 milliampere | Temporal cortex | Total six stimulations | Unknown |  |
| NCT04759092 | AD | 30 min low dose tDCS (2mA) | Left temporal lobe | 30 minutes daily for 4 months | Completed | N/A |
| NCT03288363 | AD | tDCS (DC-Stimulator Plus -Neuroconn) | Dorsolateral prefrontal cortex | 30 minutes-2 mA-2 sessions per day for 2 weeks | Recruiting | N/A |
| NCT04457973 | Pain in AD and Related Dementias | tDCS  (Constant current intensity of 2 mA | Primary motor cortex; contralateral supraorbital area | 20 minutes per session daily for 5 days | Recruiting | N/A |
| NCT04855643 | Apathy in AD | tDCS  (2mA, with 30 s ramping up and down) | Left and right dorsolateral prefrontal cortexes | 30 min at an intensity of 2mA for 6 weeks | Recruiting | N/A |
| NCT04599764 | AD at early stage | High definition DCS | Head | Anodal tDCS daily for two weeks | Active, not recruiting | N/A |
| NCT04404153 | AD,  Dementia | tDCS device model Soterix mini-CT (2 mA) | Dorsolateral prefrontal cortex | 30 minutes five times per week (Monday-Friday) for 26 weeks | Recruiting | N/A |
| NCT02227953 | Mild to Moderate AD | Yband (YDT-201N)-tDCS-2 mA | Dorsolateral prefrontal cortex | 30-minute daily in the morning for 12 weeks | Unknown | N/A |
| NCT02537496 | AD | 20 Hz rTMS treatment | bilaterally to the left and right DLPFC | 4-week repetitive course of either rTMS | Completed | N/A |
| NCT01970150 | AD | rTMS real | Left Prefrontal Cortex | 21 sessions of rTMS over the course of four weeks | Terminated (No funding) | N/A |
| NCT04263194 | AD, Late Onset | rTMS (20 Hz) | Default mode network node | Intensive phase (3 weeks of treatment, 5 days per week; Maintenance phase: 1 session of treatment every 2 weeks for 5 months (10 sessions in total) | Recruiting | N/A |
| NCT01481961 | AD at early stage | rTMS  (5 seconds of 10 Hz spaced 25 seconds) | Left dorsolateral prefrontal cortex | 2 sessions per day during 5 days per week | Completed | N/A |
| NCT03270137 | AD | rTMS at 5 Hz | Left dorsolateral prefrontal cortex | 15 rTMS sessions | Unknown | N/A |
| NCT01894620 | AD; Sleep Quality | rTMS real sham | Unknown | 4 weeks of rTMS with real coil; 4 weeks of treatment with sham coil | Completed | N/A |
| NCT01885806 | AD;  Apathy | Magnetic Stimulator Magstim Rapid 2  rTMS at 10 Hz | Left dorsolateral pre-frontal cortex | 10 consecutive sessions of rTMS; 16 minutes (20 sequencies of 6 seconds of stimulation followed by intervals of 30 seconds) | Unknown | Phase 2 |
| NCT05102045 | AD | rTMS (20 Hz) | Bilateral dorsolateral prefrontal cortex | 5 consecutive days per week for over 2 weeks | Completed | N/A |
| NCT04823819 | AD | rTMS+tDCS (2 mA) | Left dorsolateral prefrontal cortex (rTMS); Left dorsolateral prefrontal cortex (tDCS); right temporal lobe (tDCS) | 20 sessions of rTMS;  tDCS: 20 minutes, 2mA  Total duration: unclear | Completed | N/A |
| NCT00814697 | Language in AD | Magstim Rapid2 stimulator (0.5-3.5 Tesla) | Right and left dorsolateral prefrontal cortex | 4 sessions of rTMS over 2 weeks, lasting approximately 30 minutes, 2 consecutive days a week for 2 weeks | Completed | Phase 2 |
| NCT02190084 | Apathy in AD | Neurostar rTMS (10 Hz) | Dorsolateral prefrontal cortex | 20 treatment sessions for 4 weeks | Completed | Phase 4 |
| NCT01504958 | AD | rTMS with real cognitive training | Left and right parietal cortex; Left and right dorsolateral prefrontal cortex, left superior temporal gyrus | 1800 pulses of up to 20Hz per day for 6 weeks | Completed | N/A |
| NCT04294888 | AD; Mild Cognitive Impairment;  Aging | rTMS with MagPro X100 stimulator | Nodes of the Default Mode Network | Delivered at 80% of a patient's active motor threshold; Administered in an excitatory iTBS pattern | Recruiting | N/A |
| NCT03612622 | AD at the early stage | TBS | Unknown | Active TMS once daily for two weeks | Completed | N/A |
